# Supplementary material for: Structural basis for the multi-activity factor Rad5 in replication stress tolerance
Source: Nat Commun. 2021 Jan 12;12:321. doi: 10.1038/s41467-020-20538-w (PMC7804152; doi:10.1038/s41467-020-20538-w)
Supplement: Supplementary file 1 — Supplementary Information [file 41467_2020_20538_MOESM1_ESM.pdf]

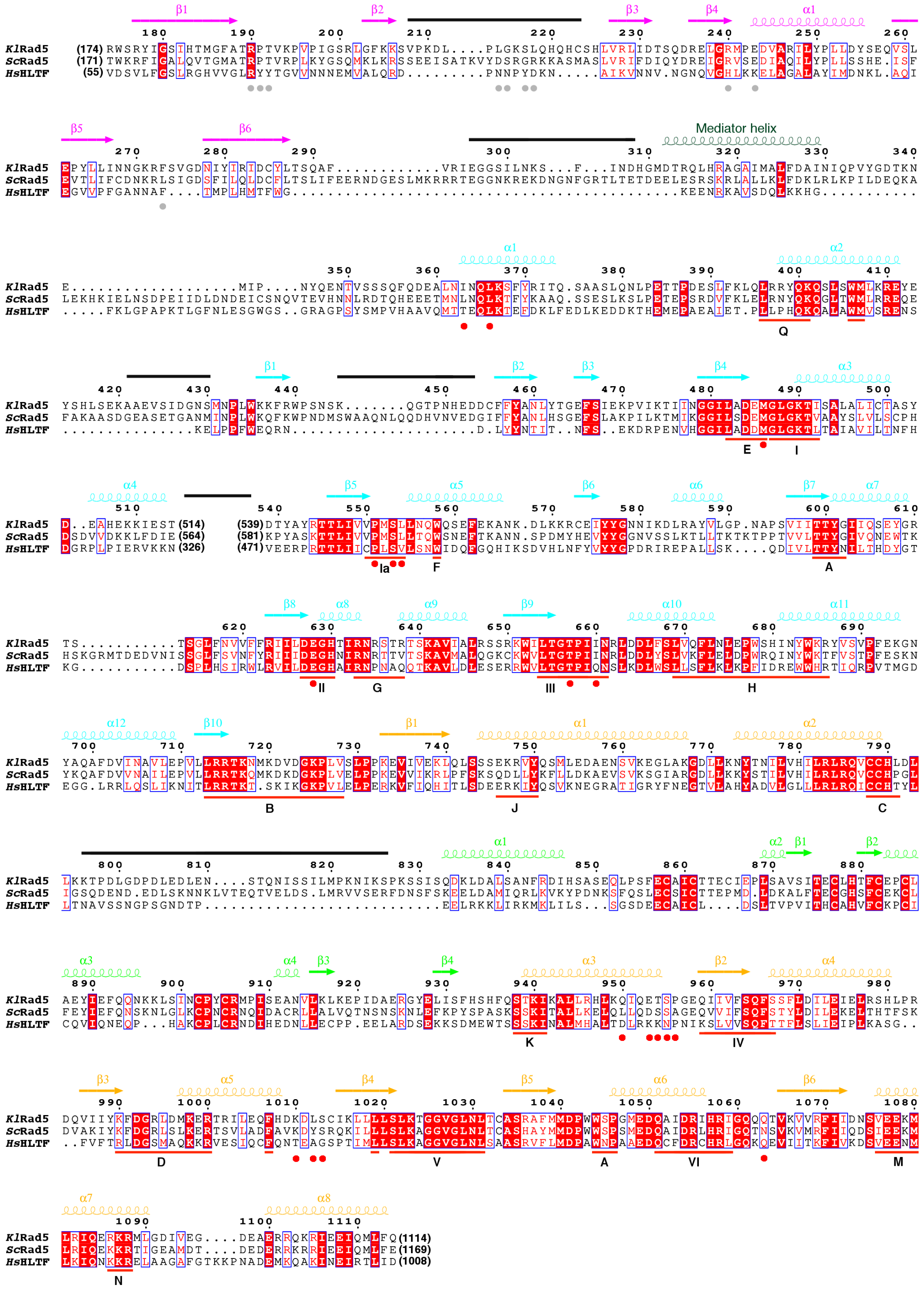

Supplementary Figure 1 Sequence alignment of Rad5 and the human HLTF (*Hs*HLTF). Residue numbers and secondary structure elements (color coded as in Figure 1a) for *Kl*Rad5 are indicated. The black lines above the sequences indicate disordered regions in our structure. Conserved motifs in the Snf2 family are indicated by the red lines below the sequences and labelled. The gray and red dots indicate residues participating in ssDNA binding in *Hs*HLTF's HIRAN domain and residues mediating interactions between the Snf2 domain lobes 1 and 2 in our structure, respectively. *Kl*Rad5 shares 46.7% sequence identity with *Sc*Rad5, 31.4% with *Hs*HLTF.

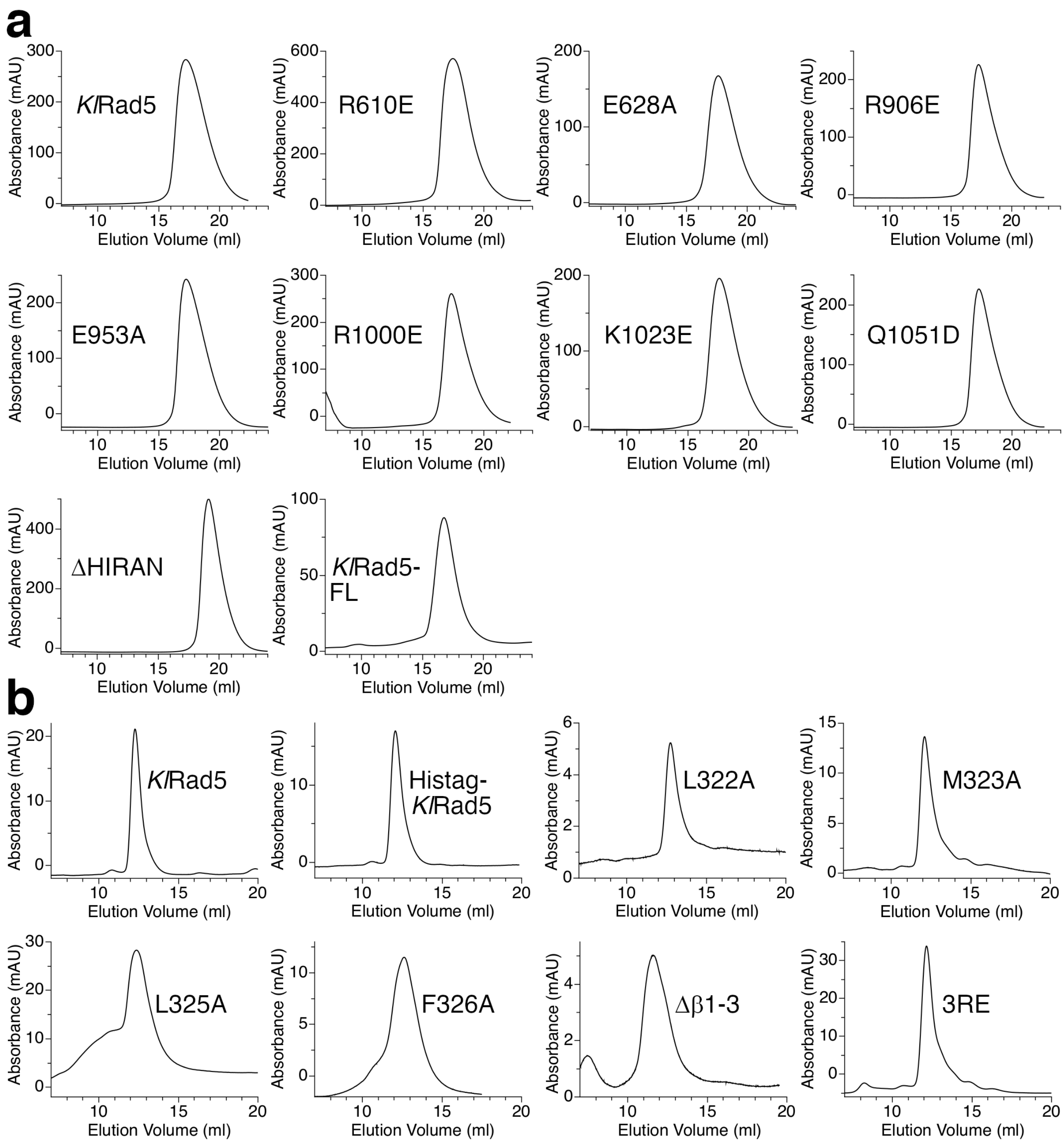

Supplementary Figure 2 Size exclusion chromatography of *Kl*Rad5 and its variants. The proteins were analyzed with a Superose 6 10/300 column (a) or a Superdex 200 10/300 column (b). The elution buffer contains 20 mM Tris (pH 7.5), 200 mM sodium chloride and 2mM DTT. *Kl*Rad5-FL, full-length *K. lactis* Rad5.

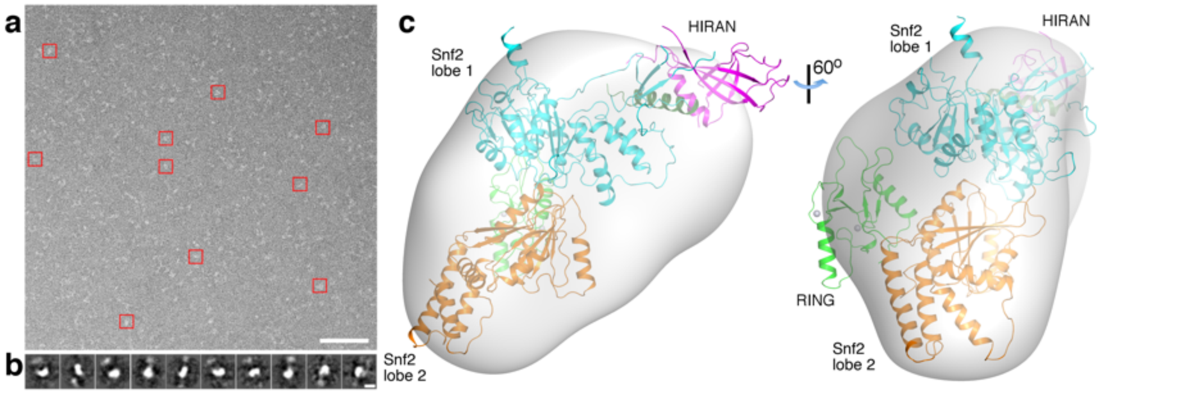

Supplementary Figure 3 Electron microscopic studies of *Kl*Rad5. (a) A representative negative staining electron microscopy (EM) image of the *Kl*Rad5 particles (red squares; bar, 40 nm). (b) Representative 2D averaging classes of the *Kl*Rad5 particles (bar, 10 nm). The 2D classification and averaging were performed once. (c) 3D reconstruction of *Kl*Rad5 from negative staining images (gray surface). The crystal structure of *Kl*Rad5 is fitted into the EM density.

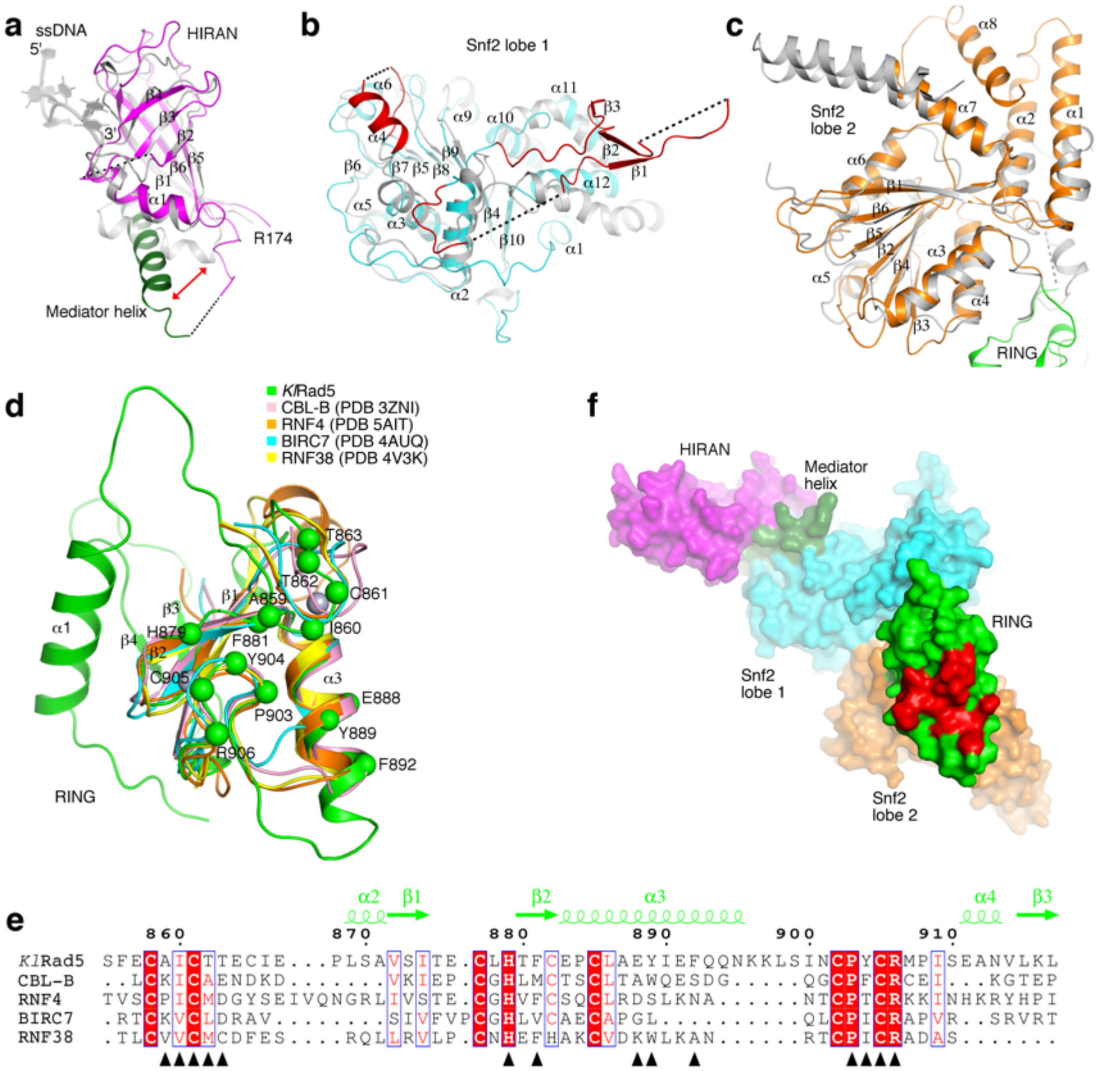

Supplementary Figure 4 Structural homologues of individual *Kl*Rad5 domains. (a) Structural alignment of the HIRAN domain and the mediator helix and equivalent regions in HLTF (PDB 4S0N, http://www.rcsb.org/structure/4S0N, gray). The ssDNA molecule bound to HLTF is shown. The red arrow indicates the large conformational difference between the mediator helix in *Kl*Rad5 and its equivalent in HLTF. Black dashed lines indicate disordered regions in our structure. (b) and (c) Structural alignments of Snf2 domain lobes 1 (b) and 2 (c) and their equivalents in the *S. cerevisiae* Snf2 (*Sc*Snf2, PDB 5X0X, http://www.rcsb.org/structure/5X0X, gray). Insertions in lobe 1 are highlighted in red. (d) Structural alignment of the RING domains in *Kl*Rad5 and selected RING ubiquitin ligases. The green spheres represent the RING domain active site residues in *Kl*Rad5, their equivalents mediate interactions with ubiquitin conjugating enzymes or ubiquitin in the selected RING ubiquitin ligases. The gray spheres represent the zinc ions. (e) Sequence alignment of the RING domains in *Kl*Rad5 and selected RING ubiquitin ligases. Sequence numbers and secondary structure elements are indicated for *Kl*Rad5. The black triangles indicate the RING domain active site residues. (f) *Kl*Rad5 presents its RING domain for efficient ubiquitin transfer reaction. The molecular surface of *Kl*Rad5 is presented. The RING domain active site residues are colored in red.

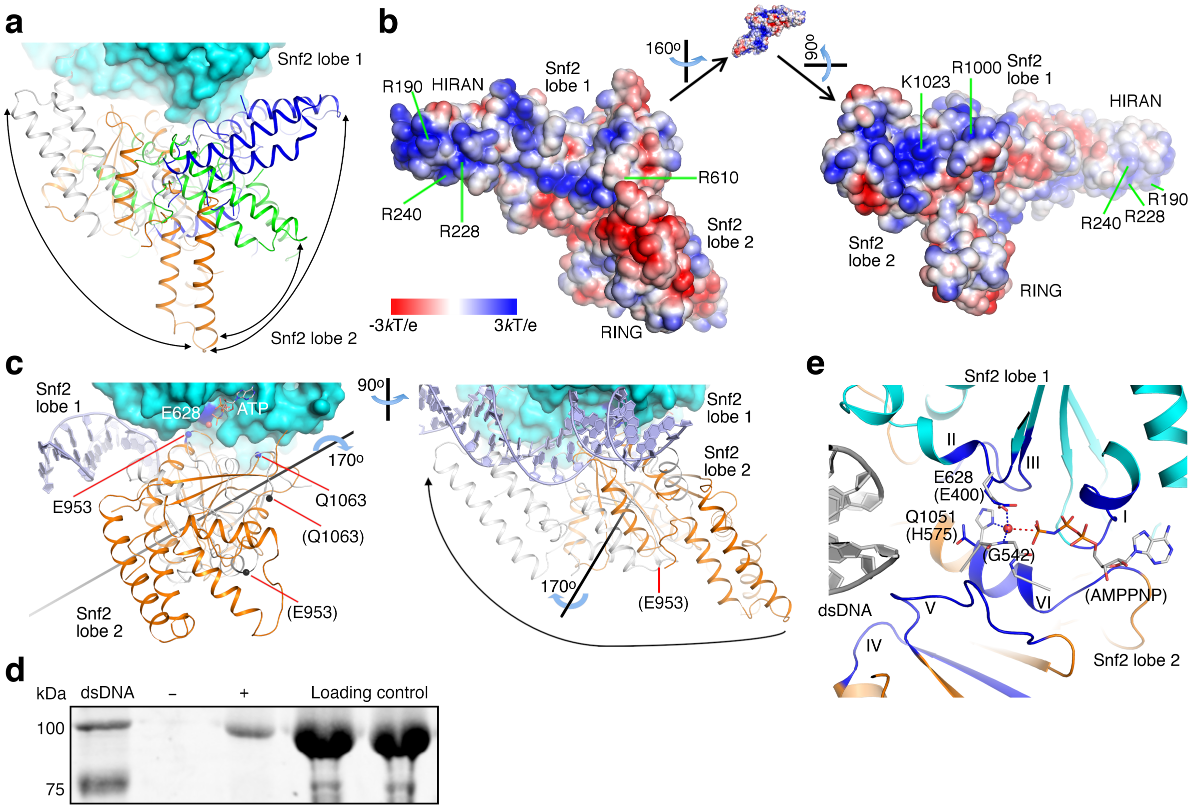

Supplementary Figure 5 dsDNA binds to *Kl*Rad5 and induces conformational changes. (a) Conformational differences of Snf2 family enzymes in the absence of DNA. The Snf2 domain lobe 2 in *Kl*Rad5 is modelled based on the DNA-free structures of the *Sulfolobus solfataricus* Rad54 (green, PDB 1Z63, http://www.rcsb.org/structure/1Z63), the zebrafish Rad54 (gray, PDB 1Z3I, http://www.rcsb.org/structure/1Z3I) or the *Myceliophthora thermophila* Snf2 (blue, PDB 5HZR, http://www.rcsb.org/structure/5HZR). The Snf2 domain in these enzymes are stabilized in ATPase-inactive conformations by interactions between their lobes 1 and 2. The arrows indicate the conformational differences between these structures and ours. (b) Electrostatic potential on the *Kl*Rad5 protein surface. (c) Modelled conformational change in *Kl*Rad5 induced by dsDNA binding. The dsDNA (light blue)-bound state (gray) is modelled based on the *Sc*Snf2-nucleosome complex structure (PDB 5X0X, http://www.rcsb.org/structure/5X0X). The 170-degree rotation of the Snf2 domain lobe 2 required to change to the dsDNA-bound state is highlighted. The black arrow in the right panel indicates the conformational change to the modelled dsDNA-bound state. The ATP molecule and the water molecule required for its hydrolysis (red sphere) are modelled based on the structure of Vasa (PDB 2DB3, http://www.rcsb.org/structure/2DB3). Positions of Glu628, Glu953 and Gln1063 observed at the Snf2 domain lobe 1-2 interface in our structure are indicated. Labels in parentheses are for the modelled dsDNA-bound state. For clarity, the RING domain is omitted in the figure. (d) SDS PAGE analysis of *Kl*Rad5 co-precipitated with biotin-labeled dsDNA. The proteins were detected with Coomassie blue staining. The same amount of protein used in the co-precipitation experiments were loaded in loading control. Two repeats of the experiment were performed, which gave similar results. (e) Glu628 and Gln1051 in *Kl*Rad5 play critical roles in its ATP hydrolysis. Positions of Snf2 domain lobes 1 and 2 in *Kl*Rad5 are modelled based on the structure of Vasa (PDB 2DB3). dsDNA (gray) is modelled based on the *Sc*Snf2-nucleosome complex structure (PDB 5X0X). Conserved motifs (blue) are labelled. The red sphere represents the water molecule required for ATP hydrolysis in Vasa. Residues coordinating it (gray for the carbon atoms) and their equivalents in *Kl*Rad5 are

highlighted. Labels in parenthesis are for Vasa. Source data for panel d is provided as Source Data file.

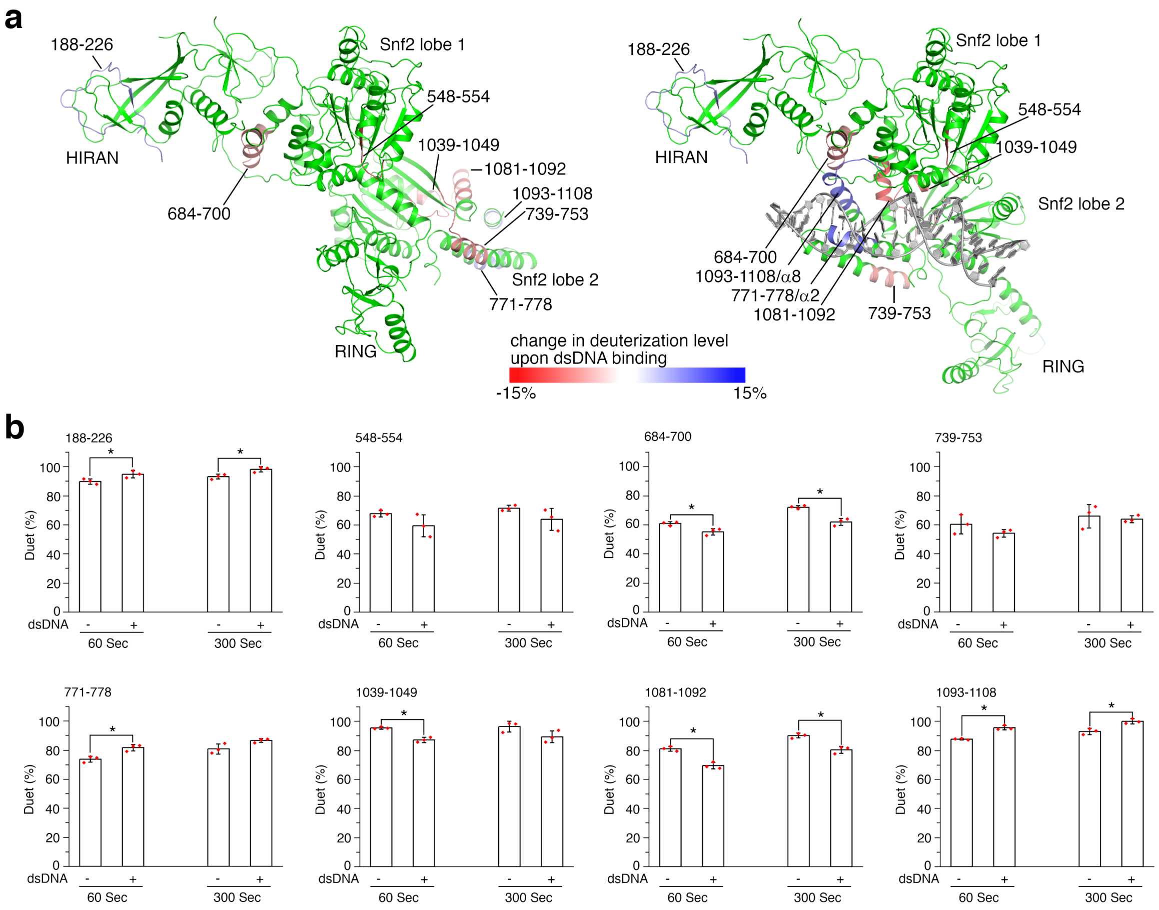

Supplementary Figure 6 Hydrogen-deuterium exchange experiments. (a) Binding to dsDNA causes changes in the deuterization level in different regions in *Kl*Rad5. The deuterization level is defined as the deuterium content divided by the maximum deuterium content, estimated by extending the D_2O incubation to 24 hours. *Kl*Rad5 peptides with significant differences in the deuterization level between experiments in the absence and presence of dsDNA are colored according to the differences in this parameter in experiments with 60-second D_2O incubation. Other regions in *Kl*Rad5 are colored in green. In the right panel, *Kl*Rad5 is presented in the modelled dsDNA (gray)-bound state. α2 and α8 in the Snf2 domain lobe 2 are indicated in the right panel. (b) Deuterization level (Duet) of *Kl*Rad5 peptides. Experiments with 60- or 300-second D_2O incubation in the absence and presence of dsDNA are presented. Data are presented as mean values +/- standard deviations of three independent experiments. The red dots indicate individual experiments. The stars indicate that the deuterium level in the presence of dsDNA is significantly different from the deuterium level in the absence of it ($p<0.05$ in the two-tailed T-test). The p values are: 0.0491 (188-226 (peptide)/60 sec (incubation time in D_2O)), 0.0244 (188-226/300 sec), 0.144 (548-554/60 sec), 0.162 (548-554/300 sec), 0.0213 (684-700/60 sec), 0.00267 (684-700/300 sec), 0.210 (739-753/60 sec), 0.688 (739-753/300 sec), 0.0113 (771-778/60 sec), 0.0589 (771-778/300 sec), 0.00191 (1039-1049/60 sec), 0.0878 (1039-1049/300 sec), 0.00200 (1081-1092/60 sec), 0.00467 (1081-1092/300 sec), 0.00107 (1093-1108/60 sec) and 0.0126 (1093-1108/300 sec). Source data for panel b is provided as Source Data file.

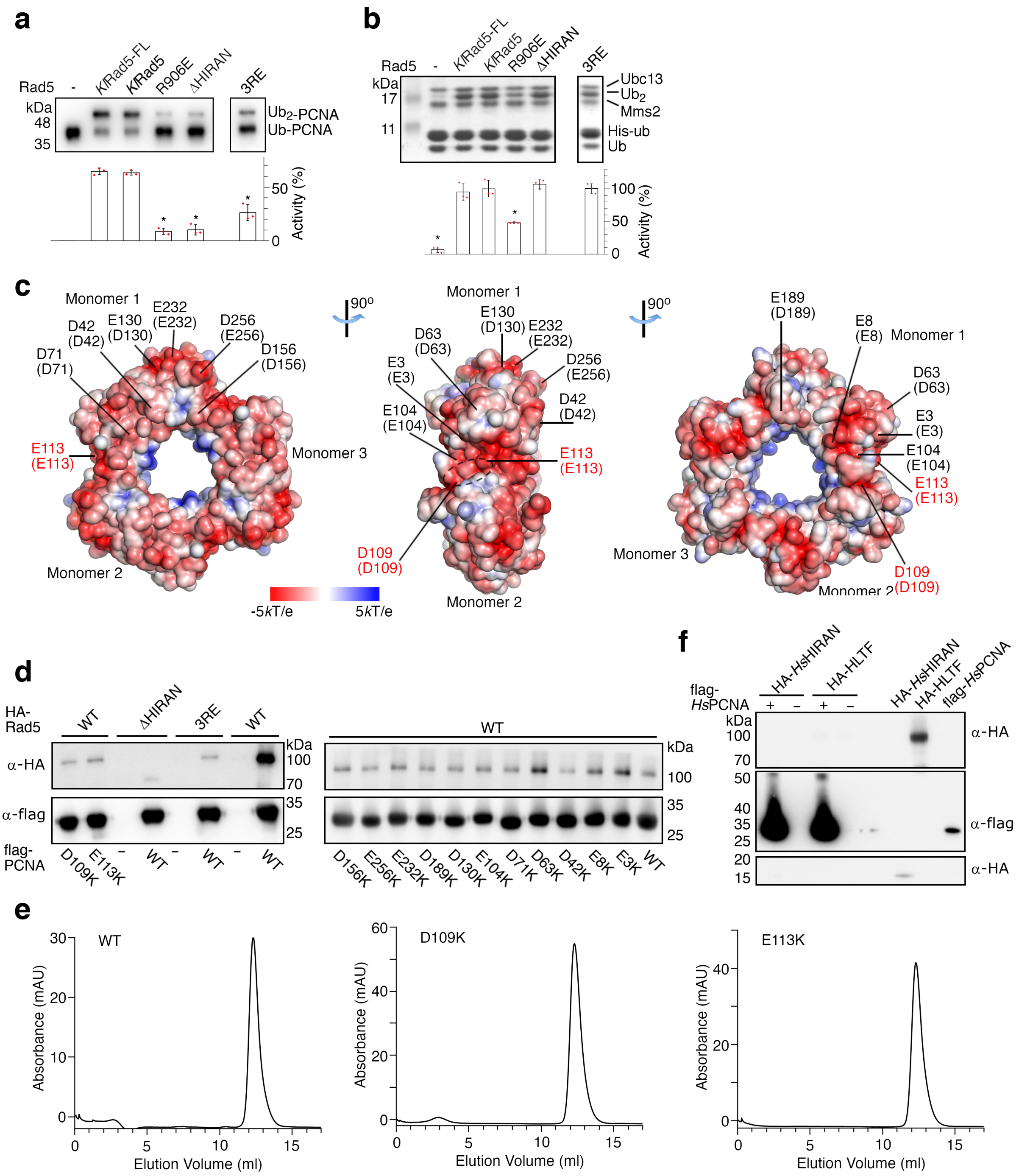

Supplementary Figure 7 The conserved and positively charged region in the HIRAN domain plays a critical role in the Rad5-PCNA interaction and the Rad5-catalyzed PCNA ubiquitination. (a) PCNA-anchored ubiquitin-chain extension by *Kl*Rad5 and its variants. Western blot analysis against the Ub-PCNA fusion protein substrate is presented. Ubiquitin with the K63R substitution is used to ensure the production of only the Ub_2-PCNA conjugate. The percentage of Ub-PCNA fusion protein ubiquitinated is used to quantify the activity (lower panel). *Kl*Rad5-FL, full-length *K. lactis* Rad5. (b) Stimulation of unanchored ubiquitin-chain formation by *Kl*Rad5 and its variants. SDS PAGE analysis of the reactions is presented. His-tagged ubiquitin with the G76C substitution and untagged ubiquitin with the K63R substitution are used in the reaction. Only Ub_2 is produced by the reaction, the amount of which is used to quantify the activity (lower panel). Activity of the wild type *Kl*Rad5 is set to be 100%. Data in the bar diagrams in panels a-b are presented as mean values +/- standard deviations of three

independent experiments. The red dots indicate individual experiments. The stars indicate activities significantly different from that of the wild type *Kl*Rad5 ($p<0.005$ in the two-tailed T-test). The p values for experiments presented in panel a are: 0.604 (*Kl*Rad5-FL), 0.0000136 (R906E), 0.0000639 (ΔHIRAN) and 0.00125 (3RE); for experiments presented in panel b are: 0.000262 (No Rad5), 0.664 (*Kl*Rad5-FL), 0.00209 (R906E), 0.447 (ΔHIRAN) and 0.965 (3RE). (c) Electrostatic potential on the outer surface of the *S. cerevisiae* PCNA (PDB 4YHR, http://www.rcsb.org/structure/4YHR). Residues equivalent to these selected for mutagenesis in the *K. lactis* PCNA are indicated. Labels in parentheses indicate equivalent residues in the *K. lactis* PCNA. For clarity, only residues in monomer 1 in the PCNA trimer are indicated. Residues potentially important for the Rad5-PCNA interaction are highlighted with red labels. The black oval in the middle panel indicates the cleft between PCNA monomers that likely mediate the Rad5-PCNA interaction. (d) Co-precipitation experiments probing the interaction between *Kl*Rad5 and the *K. lactis* PCNA and their variants. Western blot analysis of *Kl*Rad5 co-precipitated with flag-PCNA is presented. Flag-PCNA is precipitated with anti-FLAG agarose resin. In the left panel, *Kl*Rad5 variants with the 3RE substitution and the ΔHIRAN truncation are included for comparison. The exposure time of the upper left gel is longer to better image the weak bands. Three repeats of the experiments were performed, which gave similar results. (e) Size exclusion chromatography of the wild type and D109K and E113 substituted PCNA. The experiments were performed on a Superdex 200 10/300 column. The elution buffer contains 20 mM Tris (pH 7.5), 200 mM sodium chloride and 2mM DDT. (f) Co-precipitation experiments probing the interaction between *Hs*PCNA and HLTF or *Hs*HIRAN. Western blot analysis of HA-tagged HLTF or *Hs*HIRAN co-precipitated with flag-*Hs*PCNA is presented. Flag-*Hs*HIRAN is precipitated with anti-FLAG agarose resin. The right half of the blots show the input proteins. Three repeats of the experiments were performed, which gave similar results. Source data for panels a-b, d and f are provided as Source Data file.

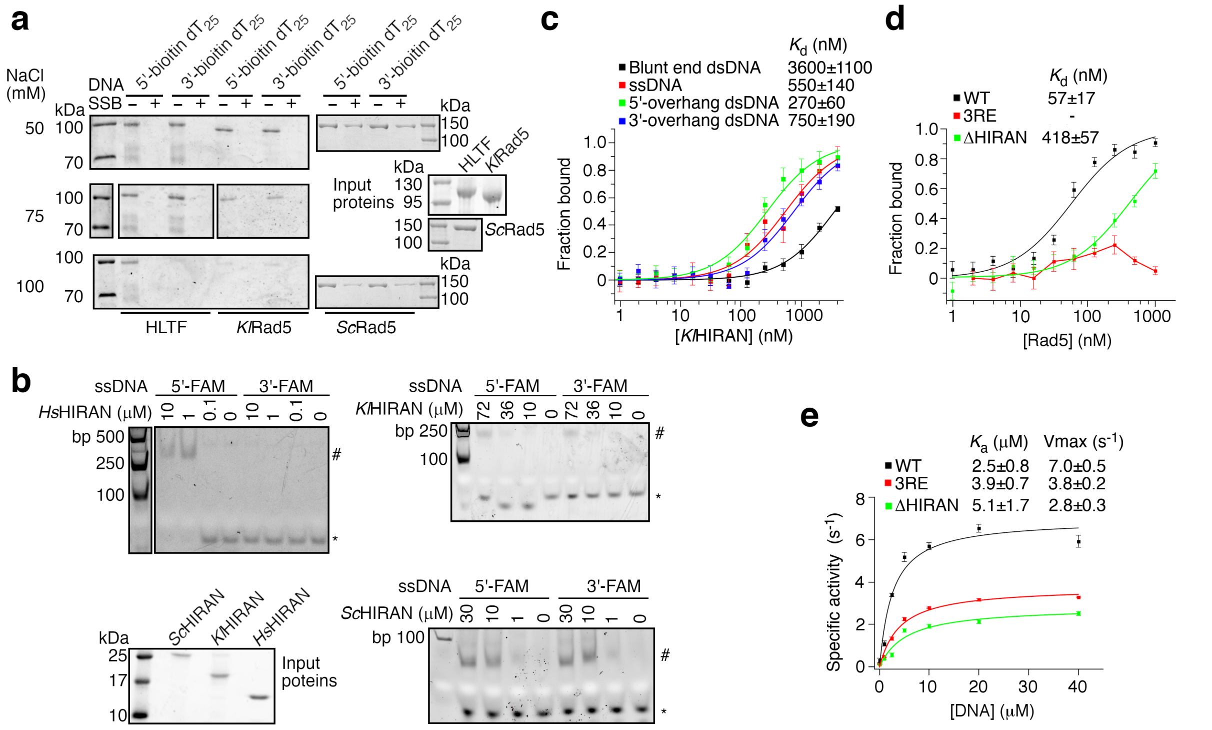

Supplementary Figure 8 *Kl*Rad5's HIRAN domain mediate interactions with DNA. (a) DNA co-precipitation experiments of HLTF, *Kl*Rad5 and *Sc*Rad5. SDS PAGE analysis of HLTF, *Kl*Rad5 or *Sc*Rad5 co-precipitated with 5'- or 3'-biotin labelled dT_{25} at different salt concentrations is shown. The proteins were detected with Coomassie blue staining. Three repeats for the experiments with HLTF and *Kl*Rad5 were performed, which gave similar results. Experiments with *Sc*Rad5 were performed once due to the limited protein amount. (b) EMSA experiments probing the interaction between ssDNA and isolated HIRAN domains. The "#" and "*" signs indicate the protein-DNA complex and the free DNA, respectively. These experiments were performed once. (c) FP experiments probing the interaction between *Kl*HIRAN and ssDNA or dsDNA with blunt ends or 5'- or 3'-overhanging ssDNA regions. (d) FP experiments probing dsDNA binding to *Kl*Rad5 with the 3RE substitution or the ΔHIRAN truncation. (e) ATPase activity of *Kl*Rad5 variants with the 3RE substitution or the ΔHIRAN truncation. Experiments with the wild type *Kl*Rad5 are included for comparison in panels d and e. Data in panels c, d and e are presented as mean values +/- standard deviations of three independent experiments. Errors in K_d, K_a and Vmax are derived from data-fitting. Source data for panels a-e are provided as Source Data file.

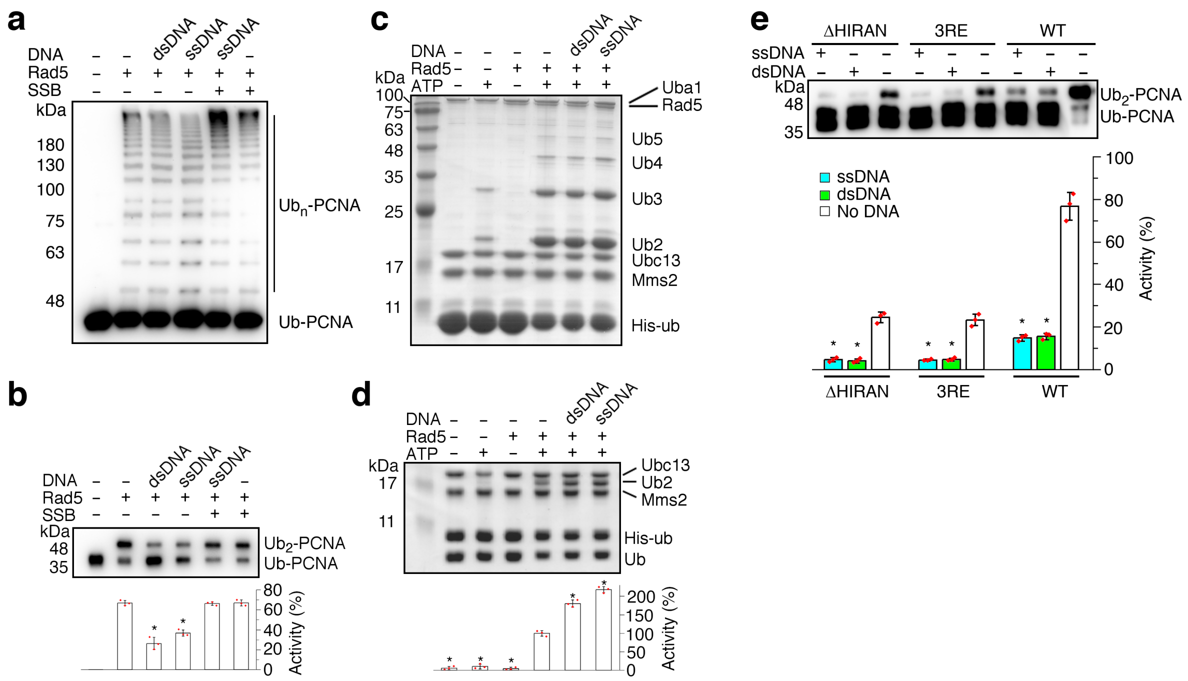

Supplementary Figure 9 DNA inhibits the *Kl*Rad5-catalyzed PCNA poly-ubiquitination but not its ubiquitin ligase activity *in vitro*. (a)-(b) PCNA-anchored ubiquitin-chain extension by *Kl*Rad5 in the presence and absence of DNA. Western blot analysis against the Ub-PCNA fusion protein substrate is presented. In panel b, ubiquitin with the K63R substitution is used to ensure production of only the Ub_2-PCNA conjugate. The percentage of Ub-PCNA fusion protein ubiquitinated is used to quantify the activity (lower panel). Three repeats of the experiment presented in panel a were performed, which gave similar results. (c)-(d) Unanchored ubiquitin-chain extension stimulated by *Kl*Rad5 in the presence and absence of DNA. SDS PAGE analysis of the reactions is presented. The proteins are detected with Coomassie staining. In panel d, His-tagged ubiquitin with the G76C substitution and untagged ubiquitin with the K63R substitution are used. Only Ub_2 is produced by the reaction, the amount of which is used to quantify the activity (lower panel). Activity of the wild type *Kl*Rad5 is set to be 100%. The experiment presented in panel c was performed once. (e) The PCNA-anchored ubiquitin-chain extension by *Kl*Rad5 variants with the 3RE substitution or the ΔHIRAN truncation can be further suppressed by DNA. The reaction with the wild type *Kl*Rad5 is included for comparison. Data in the bar diagrams in panels b, d and e are presented as mean values +/- standard deviations of three independent experiments. The red dots indicate individual experiments. The stars indicate activities significantly different from the activity measured in the absence of DNA ($p<0.005$ in the two-tailed T-test). The p values for experiments presented in panel b are: 0.000450 (dsDNA), 0.000228 (ssDNA), 0.806 (ssDNA with SSB) and 0.975 (SSB); for experiments presented in panel d are: 0.0000506 (no Rad5, DNA or ATP), 0.0000958 (ATP only), 0.0000374 (*Kl*Rad5 only), 0.000329 (with dsDNA) and 0.0000549 (with ssDNA); for experiments presented in panel e are: 0.000181 (ΔHIRAN with ssDNA), 0.000169 (ΔHIRAN with dsDNA), 0.000287 (3RE with ssDNA), 0.000329 (3RE with dsDNA), 0.0000810 (WT with ssDNA) and 0.0000868 (WT with dsDNA). Source data for panels a-e are provided as Source Data file.

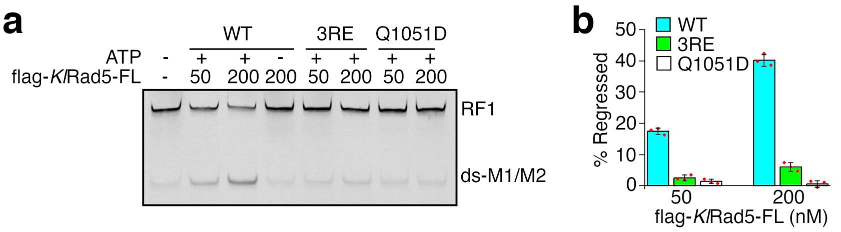

Supplementary Figure 10 Replication fork regression by *K. lactis* Rad5. (a) Replication fork regression activity of the full-length *K. lactis* Rad5 with a N-terminal flag tag (flag-*Kl*Rad5-FL) and its variants. (b) Quantification of the fork regression reactions. Data are presented as mean values +/- standard deviations of three independent experiments. The red dots indicate individual experiments. Source data for panels a-b are provided as Source Data file.

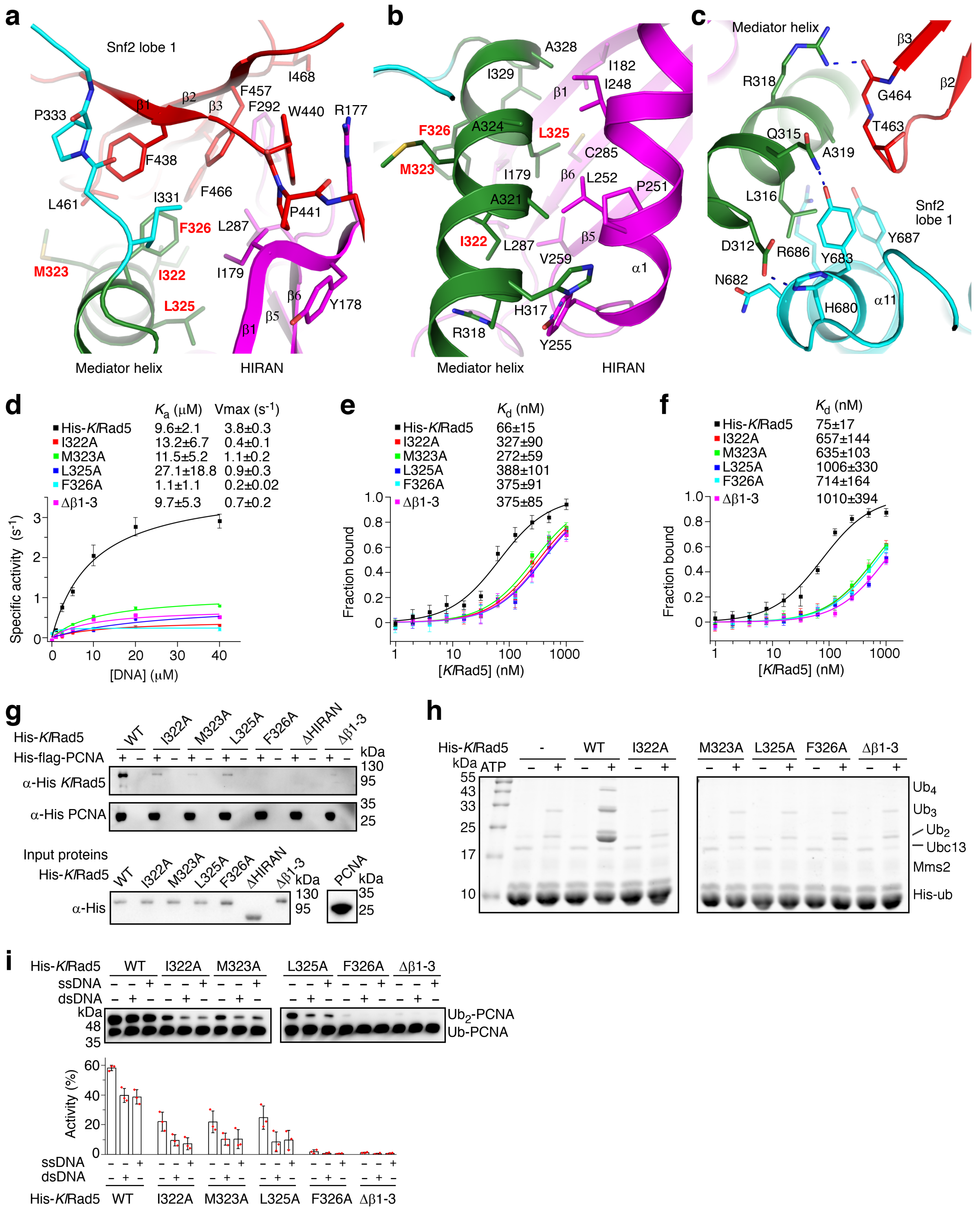

Supplementary Figure 11 The HIRAN-Snf2 interaction contributes to *Kl*Rad5's multiple activities. (a) Residues in the C-terminal half of the mediator helix form a hydrophobic core with residues in the β1-β3 insertion in Snf2 domain lobe 1 and the HIRAN domain. The β1-β3 insertion is highlighted in red. (b)-(c) Additional interactions between the mediator helix and the HIRAN domain (b) or the Snf2 domain lobe 1 (c). In panels a-c, the dashed lines indicate potential hydrogen bonds, residues selected for mutagenesis are highlighted with red labels. (d) *Kl*Rad5 variants with disrupted HIRAN-Snf2 interaction have reduced ATPase activity. (e)-(f) *Kl*Rad5 variants with disrupted HIRAN-Snf2 interaction possess reduced affinity towards dsDNA (e) and ssDNA (f). FP experiments probing DNA binding are presented. (g) *Kl*Rad5 variants with disrupted HIRAN-Snf2 interaction possess reduced

affinity towards PCNA. Western blot analysis of *Kl*Rad5 variants co-precipitated with PCNA is presented. The ΔHIRAN variant is included for comparison. Three repeats of this experiment were performed, which gave similar results. (h) *Kl*Rad5 variants with disrupted HIRAN-Snf2 interaction possess reduced activity to catalyze free ubiquitin-chain extension. SDS PAGE analysis of the ubiquitin-chain extension reaction is presented. The proteins were detected with Coomassie blue staining. Ub_2-Ub_4 indicate ubiquitin-chains with 2-4 ubiquitin moieties. This experiment was performed once. (i) *Kl*Rad5 variants with disrupted HIRAN-Snf2 interaction possess reduced activity to catalyze PCNA-anchored ubiquitin-chain extension. Western blot analysis against the Ub-PCNA fusion protein substrate is presented. Ubiquitin with the K63R substitution is used to ensure production of only the Ub_2-PCNA conjugate. The percentage of Ub-PCNA fusion protein ubiquitinated is used to quantify the activity (lower panel). *Kl*Rad5 variants used in experiments presented in panels d-i contain a N-terminal Histag. Data in panels d-f and the bar diagram in panel i are presented as mean values +/- standard deviations of three independent experiments. The red dots in the bar diagram in panel i represent individual experiments. Errors in K_a, Vmax and K_d are derived from data-fitting. Source data for panels d-i are provided as Source Data file.

Supplementary Table 1 Sequences of chemically synthesized genes

Synthesized gene fragment for the first 162 residues in *K. lactis* Rad5
atgacccagccgcagaaagccgatgaaaaaccgcgcttcttccgtgacgaagacgaaagcgtgatcaacctgaacgaaagccg cagcctgtttgtgcaggacgaggcagatgatagtgatgatggtcagggccatgtggaaagcagtagcgaggtgagtagcaacac cagcaaccacaaagagcgcttctttgagagcctgaaggagatcctgggcgaaaacatgatcagcagcagccagctgcataccct gtggagcagctatggcctgctgaaagacggcatcagcattgccgccgataaatttttgaggacaaggaactgctgagcaagagc aaaaccgagagcaaaatgccggccggcgataacatcgaggttatcgacctgagcgacgaggaaaacgacgacgagctgagtat tctgccgagcagcagccaactgagccagctgttcaccaataaacgcaccagcacccaggccggtctg
Synthesized gene for HLTF
atgagctggatgtttaaacgtgacccggtttggaaatacctgcagaccgttcaatacggcgtgcacggtaacttcccgcgtctgagc tatccgaccttcttcccgcgctttgaatttcaggacgtgattccgccggatgacttcctgacctctgatgaagaagttgatagcgttctg tttggttccctgcgcggccacgttgttggtctgcgctactataccggcgtagtgaataacaacgaaatggttgcgctgcagcgtgatc ctaacaacccgtacgataaaaacgcgattaaagttaacaacgtgaacggtaaccaggttggccacctgaaaaaagaactggcagg cgcgctggcatacattatggataacaaactggctcagatcgaaggtgtagttccgttcggtgctaacaatgcgtttaccatgccgctg cacatgaccttctggggtaaagaagaaaaccgtaaagccgtgtctgatcaactgaaaaaacacggctttaaactgggcccggcac caaaaaccctgggcttcaacctggaatccggctggggtagcggtcgtgcaggtccgtcttacagcatgccggttcatgcagctgtt cagatgaccaccgagcagctgaaaactgagttcgataaactgttcgaagacctgaaagaagatgacaaaacccatgaaatggag ccggctgaagcgattgaaactccgctcctgccgcaccagaaacaggcgctggcctggatggttagccgtgaaaacagtaaagaa ctgccgccgttctgggaacagcgtaacgacctgtactataacaccatcactaatttctccgaaaaagaccgccctgaaaatgtgcac ggcggcatcctggctgatgacatgggtctgggtaaaaccctgaccgcaatcgctgtaatcctgaccaacttccatgatggccgtcc actgccgatcgaacgtgtgaaaaagaacctgctgaaaaaagaatataacgttaacgatgattctatgaaactgggtggtaataacac ttctgagaaagccgatggcctgagtaaagacgctagccgttgcagcgaacagccgagcatctctgacatcaaagagaaatccaaa ttccgtatgagcgaactgtcgagcagccgcccgaaacgtcgtaaaaccgctgtgcagtacattgaatcgtctgattcggaggaaatt gaaacttctgaactgccacagaaaatgaaaggcaaactgaaaaacgtgcagagtgaaaccaaaggtcgtgctaaagcgggttcct ctaaagtgattgaagatgttgcattcgcgtgcgcgctgaccagctccgttccgactaccaaaaagaaaatgctgaaaaagggcgct tgcgcggttgaaggtagcaagaaaaccgatgttgaagaacgtccgcgtaccaccctgattatttgtccgctgagcgttctgtccaac tggatcgaccagtttggccagcatattaaatccgatgtgcacctgaacttctatgtttattatggtccagaccgcatccgtgaaccggc tctgctgtctaaacaagacatcgttctgaccacctataacatcctgacccatgactacggcactaaaggtgactcaccgctgcactct attcgttggctgcgcgttatcctggatgaaggtcacgcaatccgtaacccaaacgcgcagcagactaaagccgtgctggatctgga atccgaacgccgttgggtgctgaccgggaccccgattcaaaactccctgaaagatctgtggtctctgctgtcctttctgaaattgaaa ccgttcattgaccgtgaatggtggcaccgtaccatccagcgtccggtgaccatgggtgatgaaggcggtctgcgccgtctgcagtc tctgatcaaaaacattactctgcgtcgcactaaaacctccaaaattaaaggtaaaccggttctggaactgccggaacgcaaagtgttc attcagcacattaccctgagcgacgaagaacgtaaaatctaccagagcgttaaaaatgaaggtcgtgctaccatcggccgctacttc aacgaaggcaccgttctggcgcattacgctgatgtgctgggcctgctgctgcgtctgcgtcagatttgttgccacacctatttactga ccaacgcggtaagcagcaacgggccgtccgcgttttctctgggtaatgataccccctgaagaactgcgtaaaaagctgattcgcaaa atgaaactgatcctgagtagcggttctgatgaagaatgcgccatttgtttagatagcctgacggttccggtgattactcactgcgcaca cgttttctgtaaaccatgcatctgccaggtaatccagaacgaacagccgcatgctaaatgcccgctctgccgcaacgatatccatga agataacctgctggaatgcccgccggaagaactggcccgtgacagcgagaaaaaatctgatatggaatggacctcttcttccaaa atcaacgcactgatgcacgcactgactgacctgcgtaaaaagaacccgaacatcaaatctcttgtggttagccagttcaccaccttc ctgtctctgattgagatcccgctgaaagcctccggctttgtttttacccgcctggatggctccatggcccagaaaaaacgcgttgaaa gcatccagtgcttccagaacaccgaagccggtagcccgaccatcatgctgctgtctctgaaagcgggcggcgttggtctgaacct gagcgcggctagccgtgtttttctgatggacccggcgtggaacccggcagcggaagaccagtgctttgatcgttgtcaccgcctg ggtcaaaaacaggaagttatcatcaccaaattcatcgtgaaagactctgttgaagaaaacatgctgaaaatccagaacaaaaaacgt gaactggcggcaggtgcgtttggtaccaaaaaaccgaacgcggatgaaatgaaacaggctaaaattaacgaaatccgtaccctga ttgatctgtaa

Supplementary Table 2 Yeast strains used in this study

Strain	Genotype
334	*MATα pep4-3prb1-1122 ura3-52 leu2-3,112 reg1-501 gal1*
X3826-8D	*MATa pol30::URA3 leu2::YIp128-HisPOL30[LEU2]*
X8077-5A	*pol30::URA3 leu2::YIp128-HisPOL30[LEU2] rad5-3RE*
X3824-1A	*pol30::URA3 leu2::YIp128-HisPOL30[LEU2] rad5Δ::KAN*
T585	*MATa Rad5-TAP::HIS3*
T2130-4	*MATa rad5-3RE-TAP::HIS3*
T2129-5	*MATa rad5-3RE*
T638	*MATα rad5Δ::KAN*
Z361	*MATα rad5Δ-AA*
T1646	*MATα rad5-QD::KAN*
T1712	*MATα rad5-I916A*
pJ69-4	*TRP1-901 leu2-3, 112 ura3-52 his3-200 gal4Δ gal80Δ LYS::GAL1-HOS3 GAL2-ADE2 met2::GAL7-lacZ*

Supplementary Table 3 Sequences of primers used for plasmid construction

Primer	Sequence
*Kl*Rad5, 5' primer	CAAGCACATATGGAATCACCTATGTCATCTGACAAG
*Kl*Rad5, 3' primer	CTTGAGGCGGCCGCCTATTGAAACAACATTTGGATTTC
*Kl*Rad5 R190E substitution, 5' primer	GCTACAGCGCCAACCGTAAAACCAGTT
*Kl*Rad5 R190E substitution, 3' primer	GGTTGGCGCTGTAGCGAATCCCATAGT
*Kl*Rad5 R228E/R240E substitution, 5' primer	GAATTAATCGATACCTCACAGGATAGAGAACTTGGTGAAATGCCTG AAGATGTTGCA
*Kl*Rad5 R228E/R240E substitution, 3' primer	TTCACCAAGTTCTCTATCCTGTGAGGTATCGATTAATTCAACTAAAT GCGAACAGTG
*Kl*Rad5 R610E substitution, 5' primer	TATGGGGAAACCTCAACATCGGGATTATTC
*Kl*Rad5 R610E substitution, 3' primer	TGAGGTTTCCCCATATTCGCTCTGAATTAT
*Kl*Rad5 E628A substitution, 5' primer	TTGGATGCAGGTCATACGATTAGAAATAGA
*Kl*Rad5 E628A substitution, 3' primer	ATGACCTGCATCCAATATTATTCTAAAGAA
*Kl*Rad5 R906E substitution, 5' primer	TATTGTGAAATGCCGATCTCAGAAGCTAAT
*Kl*Rad5 R906E substitution, 3' primer	CGGCATTTCACAATATGGACAGTTGATGGA
*Kl*Rad5 R953A substitution, 5' primer	CAAATACAAGCAACTTCTCCTGGTGAACAA
*Kl*Rad5 R953A substitution, 3' primer	AGGAGAAGTTGCTTGTATTTGCTTGAGATG
*Kl*Rad5 R1000E substitution, 5' primer	AAGGAAGAGACTAGAATCTTGGAACAGTTC
*Kl*Rad5 R1000E substitution, 3' primer	TCTAGTCTCTTCCTTCATATCTAACCGTCC
*Kl*Rad5 K1023E substitution, 5' primer	TCGTTGGAAACTGGAGGTGTGGGATTAAAT
*Kl*Rad5 K1023E substitution, 3' primer	TCCAGTTTCCAACGACAAAAGTAATAACTT
*Kl*Rad5 Q1051D substitution, 5' primer	GAAGATGATGCGATTGATAGAATACAT
*Kl*Rad5 Q1051D substitution, 3' primer	AATCGCATCATCTTCCATACCTGGAGA
HA-*Kl*Rad5, 5' primer	TACCCATACGACGTCCCAGACTACGCTGAATCACCTATGTCATCTG AC
HA-*Kl*Rad5, 3' primer	AGCGTAGTCTGGGACGTCGTATGGGTACATATGTATATCTCCTTCTT A
His-*Kl*Rad5, 5' primer	CAAGCACATATGGAATCACCTATGTCATCTGACAAG
His-*Kl*Rad5, 3' primer	CTTGAGGCGGCCGCCTATTGAAACAACATTTGGATTTC
His-*Kl*Rad5, I322A substitution, 5' primer	GGTGCAGCAATGGCCCTTTTTGATGCCATTAAT
His-*Kl*Rad5, I322A substitution, 3' primer	GGCCATTGCTGCACCCGCTCTATGCAGCTG
His-*Kl*Rad5, M323A substitution, 5' primer	GCAATAGCGGCCCTTTTTGATGCCATTAATATT
His-*Kl*Rad5, M323A substitution, 3' primer	AAGGGCCGCTATTGCACCCGCTCTATGCAGCTG
His-*Kl*Rad5, L325A substitution, 5' primer	ATGGCCGCTTTTGATGCCATTAATATTCAGCCA
His-*Kl*Rad5, L325A substitution, 3' primer	ATCAAAAGCGGCCATTATTGCACCCGCTCTATG

His-*Kl*Rad5, F326A substitution, 5' primer	GCCCTTGCTGATGCCATTAATATTCAGCCAGTT
His-*Kl*Rad5, F326A substitution, 3' primer	GGCATCAGCAAGGGCCATTATTGCACCCGCTCT
*Kl*Rad5 Δβ1-3, 5' primer	GGTAGCGGTAGCGGAGGTATTTGGCGGATGAG
*Kl*Rad5 Δβ1-3, 3' primer	GCTACCGCTACCTTCTCTTTTGAGCATCCATGA
K. lactis Rad5 354-1114, 5' primer	CACTGTTCATATGTCCCAGTTTCAGGATGAAGCTTTG
K. lactis Rad5 354-1114, 3' primer	CTTGAGGCGGCCGCCTATTGAAACAACATTTGGATTTC
HA-*Kl*Rad5 ΔHIRAN, 5' primer	TACCCATACGACGTCCCAGACTACGCTTCCCAGTTTCAGGATGAAGCT
HA-*Kl*Rad5 ΔHIRAN, 3' primer	AGCGTAGTCTGGGACGTCGTATGGGTACATATGGCTGCCGCGCGGCAC
Flag-*Kl*Rad5-FL, 5' primer	GGAGATATACATATGGACTACAAAGACGATGACGACAAGATGACCCAGCCGCAGAAAGCCGATGAAAAACCG
Flag-*Kl*Rad5-FL, 3' primer	CTTTCTGCGGCTGGGTCATCTTGTCGTCATCGTCTTTGTAGTCCATATGTATATCTCCTTCTTAAAG
*Kl*HIRAN, 5' primer	TAAGAAGGAGATATACATATGCGATGGTCTCGTTACATTGGG
*Kl*HIRAN, 3' primer	TGCATCTCCCGTGATGCACTCATTTTTGGTGTCTCCGTA
*Kl*Mms2, 5' primer	TAAGAAGGAGATATACATATGTCTAAAGTACCAAGAAGT
*Kl*Mms2, 3' primer	ACCCTTGGCAAAGCACCCCTCAAACGTCGTTCCCTC
*Kl*Ubc13, 5' primer	TAAGAAGGAGATATACATATGGCAGCATTACCCAAGAGA
*Kl*Ubc13, 3' primer	GTGGTGGTGGTGGTGCTCGAGTTATTCTTTTGCTGGTTC
*Sc*HIRAN, 5' primer	TAAGAAGGAGATATACATATGACTTGGAAAAGATTTATAGGT
*Sc*HIRAN, 3' primer	TGCATCTCCCGTGATGCATAAATCAATGATTTCGGGGTC
*Sc*Uba1, 5' primer	AGTGGTGCTAGCGCCGCCGGAGAAATCGATGAAAG
*Sc*Uba1, 3' primer	GTGCGTCTCGAGTCATAGATGAATGGTAATGAAAG
S. cerevisiae Ubiquitin (*Sc*Ub), 5' primer	TAAGAAGGAGATATACATATGCAGATTTTCGTCAAGACT
*Sc*Ub, 3' primer	ACCCTTGGCAAAGCACCCACCTCTTAGCCTTAGCAC
*Sc*Ub K63R substitution, 5' primer	ATTCAGAGGGAGTCCACCTTACATCTTGTG
*Sc*Ub K63R substitution, 3' primer	GGTGGACTCCCTCTGAATGTTGTAATCAGACAG
His-*Sc*Ub, 5' primer	GCGCGGCAGCCATATGATGCAGATTTTCGTCAAGACT
His-*Sc*Ub, 3' primer	GGTGGTGGTGCTCGAGCTAACCACCTCTTAGCCTTAG
His-*Sc*Ub G76C substitution, 5' primer	AGGCTAAGAGGTTGTTAGCTCGAGCACCACCAC
His-*Sc*Ub G76C substitution, 3' primer	GTGCTCGAGCTAACAACCTCTTAGCCTTAGCAC
His-flag-Ub-*Kl*PCNA, 5' primer 1	CCGCGCGGCAGCCATGATTACAAGGATGACGACGATAAGATGCAGATTTTCGTCAAG
His-flag-Ub-*Kl*PCNA, 3' primer 1	TTTACCCGGGATCTGCAC ACCACCTCTTAGCCTTAGCAC
His-flag-Ub-*Kl*PCNA, 5' primer 2	GTGCAGATCCCGGGTAAAATGCTAGAAGCTAAATTCACA
His-flag-Ub-*Kl*PCNA, 3' primer 2	GTGGTGGTGCTCGAGTCATCATTCCTCTTCATC
His-*Kl*PCNA, 5' primer	CCGCGCGGCAGCCATATGCTAGAAGCTAAATTC
His-*Kl*PCNA, 3' primer	GTGGTGGTGCTCGAGTCATCATTCCTCTTCATC
His-flag-*Kl*PCNA, 5' primer	CCGCGCGGCAGCCATGATTACAAGGATGACGACGATAAGATGCTAGAAGCTAAATTCAC
His-flag-*Kl*PCNA, 3' primer	CTTATCGTCGTCATCCTTGTAATCATGGCTGCCGCGCGG

His-flag-*Kl*PCNA, E3K substitution, 5’ primer	ATGCTAAAAGCTAAATTCACAGAAGCTGGG
His-flag-*Kl*PCNA, E3K substitution, 3’ primer	TTTAGCTTTTAGCATCTTATCGTCGTCATC
His-flag-*Kl*PCNA, E8K substitution, 5’ primer	TTCACAAAAGCTGGGTTATTCAAAAGAATT
His-flag-*Kl*PCNA, E8K substitution, 3’ primer	CCCAGCTTTTGTGAATTTAGCTTCTAGCAT
His-flag-*Kl*PCNA, D42K substitution, 5’ primer	GTCGATAAATCTCGTGTCCTCCTTGTCTCT
His-flag-*Kl*PCNA, D42K substitution, 3’ primer	ACGAGATTTATCGACGGCTTGTGCAGCGAT
His-flag-*Kl*PCNA, D63K substitution, 5’ primer	AGAGCGAAAAGAAATGTCGTGCTAGGTTGC
His-flag-*Kl*PCNA, D63K substitution, 3’ primer	ATTTCTTTTCGCTCTAAACTCTTCAAATGC
His-flag-*Kl*PCNA, D71K substitution, 5’ primer	GGTTGCAAATTGACGAACTTGAGTAAGATT
His-flag-*Kl*PCNA, D71K substitution, 3’ primer	CGTCAATTTGCAACCTAGCACGACATTTCT
His-flag-*Kl*PCNA, E104K substitution, 5’ primer	TTATTCAAAGATACAAAAAGAGACCGTGTC
His-flag-*Kl*PCNA, E104K substitution, 3’ primer	TGTATCTTTGAATAAGATTAGAATAGAATC
His-flag-*Kl*PCNA, D109K substitution, 5’ primer	AAAAGAAAACGTGTCAGTGAATACTCTTTA
His-flag-*Kl*PCNA, D109K substitution, 3’ primer	GACACGTTTTCTTTTTGTATCTTCGAATAA
His-flag-*Kl*PCNA, E113K substitution, 5’ primer	GTCAGTAAATACTCTTTAAAATTGATGGAA
His-flag-*Kl*PCNA, E113K substitution, 3’ primer	AGAGTATTTACTGACACGGTCTCTTTTTGT
His-flag-*Kl*PCNA, D130K substitution, 5’ primer	ATCTCTAAAATGAAATATGAATCCTATATT
His-flag-*Kl*PCNA, D130K substitution, 3’ primer	TTTCATTTTAGAGATCTCCAAGAAATCTGT
His-flag-*Kl*PCNA, D156K substitution, 5’ primer	TTGAGTAAATCTATTAACATCTTAGTCACT
His-flag-*Kl*PCNA, D156K substitution, 3’ primer	AATAGATTTACTCAATTGACTTAAGTCACG
His-flag-*Kl*PCNA, D189K substitution, 5’ primer	GATTTGAAACATCCTGAAAGTTCAATTAAA
His-flag-*Kl*PCNA, D189K substitution, 3’ primer	AGGATGTTTCAAATCGGTGAAAGGTTTCAC
His-flag-*Kl*PCNA, E232K substitution, 5’ primer	TCGGAAAAAGCGCCAGCCCTTTTCCAATTC
His-flag-*Kl*PCNA, E232K substitution, 3’ primer	TGGCGCTTTTTCCGATAATTTTATAGTGAT
His-flag-*Kl*PCNA, E256K substitution, 5’ primer	TTCGATAAAGAGGAATGATGACTCGAGCAC
His-flag-*Kl*PCNA, E256K substitution, 3’ primer	TTCCTCTTTATCGAACTTTGGAGCCAAATA
His-flag-*Hs*PCNA, 5’ primer	CCGCGCGGCAGCCATGATTACAAGGATGACGACGATAAGATGTTC GAGGCGCGCCTGGTCCAG
His-flag-*Hs*PCNA, 3’ primer	GTGGTGGTGGTGGTGCTCGAGCTAAGATCCTTCTTCATCCTCGAT
HLTF, 5’ primer	AGAAGGAGATATACATATGCCGCGTCTGAGCTATCCGACC
HLTF, 3’ primer	GGTGGTGGTGCTCGAGTCATTACAGATCAATCAGGGT

HA-HLTF, 5' primer	TACCCATACGACGTCCCAGACTACGCTCCGCGTCTGAGCTATCCGACC
HA-HLTF, 3' primer	AGCGTAGTCTGGGACGTCGTATGGGTAATGGCTGCCGCGCGGCACCAGGCC
HA-*Hs*HIRAN, 5' primer	GTGCCGCGCGGCAGCCATATGTACCCATACGACGTCCCAGACTACGCTGTTGATAGCGTTCTGTTTGGTTCC
HA-*Hs*HIRAN, 3' primer	GTGGTGGTGGTGGTGCTCGAGTCAGAAGCCCAGGGTTTTTGGTGCCGG
*Sc*Rad5 expression construct, 5' primer	CTCGGATCCAATATGGACTACAAAGACGATGACGACAAGATGAGTCATATTGAACAGGAAGAAAGGAAG
*Sc*Rad5 expression construct, 3' primer	GTTCAATATGACTCATCTTGTCGTCATCGTCTTTGTAGTCCATATTGGATCCGAGCTCGGTACCAAG
*Sc*Rad5 R187E substitution, 5' primer	CTGGTATGGCTACCGAACCCACCGTCAGGCCCTTGAAGTAC
*Sc*Rad5 R187E substitution, 3' primer	GGCCTGACGGTGGGTTCGGTAGCCATACCAGTGACTTGCAAAGC
*Sc*Rad5 R229E/R241E substitution, 5' primer	ATGGCTAGTTTGGTAGAAATTTTTGATATCCAATATGATAGAGAAATTGGCGAAGTTTCGGAAGACATTGCTCAAATAC
*Sc*Rad5 R229E/R241E substitution, 3' primer	ATGTCTTCCGAAACTTCGCCAATTTCTCTATCATATTGGATATCAAAAATTTCTACCAAACTAGCCATGGACGCTTTC
*Sc*3RE-F	CACTGGTATGGCTACCGA
*Sc*3RE-R	CAATGTCTTCCGAAACTTC
*Sc*Rad5-F	TCACAGCTGATGAGTCATATTGAACAGGAAG
*Sc*Rad5-R	CTGGTCGACCTATTCAAACAGCATCTGGAT

Supplementary Table 4 Sequences of oligonucleotides used in activity experiments

Name	Sequence (5’-3’)
D1	TCGGATCCTCTAGACAGCTCCATGCATGGAGCTGTCTAGAGGATCCGA
D2a	ACCAGTGCCAGTGAT
D2b	ATCACTGGCACTGGT
D3a	CAACGTCATAGACGATTACATTGCTACATGGAGCTGTCTAGAGGATCCGA
D3b	TCGGATCCTCTAGACAGCTCCATGTAGCAATGTAATCGTCTATGACGTTG
D3c	TCGGATCCTCTAGACAGCTCCATGT
D3d	AGCAATGTAATCGTCTATGACGTTG
S1	TTTTTTTCGTCTTCGGCAATTTTTT
M1	GACGCTGCCGAATTCTACCAGTGCCTTGCTAGGACATCTTTGCCCACCTGCAGGTTCA CCCTCTACGTCTTATCATGATA
M2	TATCATGATAAGACGTAGAGGGTGAACCTGCAGGTGGGCAAAGATGTCCCAGCAAGG CACTGGTAGAATTCGGCAGCGTC
M3	TATCATGATAAGACGTAGAGGGTGAACCTGCAGGTGGGCAAAGATGTCC
M4	GGACATCTTTGCCCACCTGCAGGTTCACCCTCTACGTCTTATCATGATA
M5	TATCATGATAAGACGTAGAGGGTG
M6	CACCCTCTACGTCTTATCATGATA
