# Supplementary material for: Structural basis for the multi-activity factor Rad5 in replication stress tolerance
Source: Nat Commun. 2021 Jan 12;12:321. doi: 10.1038/s41467-020-20538-w (PMC7804152; doi:10.1038/s41467-020-20538-w)
Supplement: Supplementary file 3 — Reporting Summary [file 41467_2020_20538_MOESM3_ESM.pdf]

nature research

Corresponding author(s): Song Xiang

Last updated by author(s): Nov 27, 2020

Reporting Summary

Nature Research wishes to improve the reproducibility of the work that we publish. This form provides structure for consistency and transparency in reporting. For further information on Nature Research policies, see our Editorial Policies and the Editorial Policy Checklist.

Statistics

For all statistical analyses, confirm that the following items are present in the figure legend, table legend, main text, or Methods section.

n/a	Confirmed	
☐	☒	The exact sample size (*n*) for each experimental group/condition, given as a discrete number and unit of measurement
☐	☒	A statement on whether measurements were taken from distinct samples or whether the same sample was measured repeatedly
☐	☒	The statistical test(s) used AND whether they are one- or two-sided<br>*Only common tests should be described solely by name; describe more complex techniques in the Methods section.*
☒	☐	A description of all covariates tested
☒	☐	A description of any assumptions or corrections, such as tests of normality and adjustment for multiple comparisons
☐	☒	A full description of the statistical parameters including central tendency (e.g. means) or other basic estimates (e.g. regression coefficient) AND variation (e.g. standard deviation) or associated estimates of uncertainty (e.g. confidence intervals)
☐	☒	For null hypothesis testing, the test statistic (e.g. *F*, *t*, *r*) with confidence intervals, effect sizes, degrees of freedom and *P* value noted<br>*Give P values as exact values whenever suitable.*
☒	☐	For Bayesian analysis, information on the choice of priors and Markov chain Monte Carlo settings
☒	☐	For hierarchical and complex designs, identification of the appropriate level for tests and full reporting of outcomes
☒	☐	Estimates of effect sizes (e.g. Cohen's *d*, Pearson's *r*), indicating how they were calculated

Our web collection on statistics for biologists contains articles on many of the points above.

Software and code

Policy information about availability of computer code

Data collection	HKL2000
Data analysis	EMAN 2.1, COOT 0.8.9.2, O 14.0.0, PHENIX 1.13_2998, Dali server, QTIPLOT 1.0.0-rc3, DYNAMICS V6, PROTEOME DISCOVERER, XCALIBUR, HDExaminer.

For manuscripts utilizing custom algorithms or software that are central to the research but not yet described in published literature, software must be made available to editors and reviewers. We strongly encourage code deposition in a community repository (e.g. GitHub). See the Nature Research guidelines for submitting code & software for further information.

Data

Policy information about availability of data

All manuscripts must include a data availability statement. This statement should provide the following information, where applicable:

- Accession codes, unique identifiers, or web links for publicly available datasets
- A list of figures that have associated raw data
- A description of any restrictions on data availability

The structure factors and coordinates for the native and mercury-derivatized KlRad5 crystals have been deposited into the Protein Data Bank, with the accession codes 6L8N (http://www.rcsb.org/structure/6L8N) and 6L8O (http://www.rcsb.org/structure/6L8O), respectively. The source data underlying Figs 2d-e, 3a-c, 3f-g, 4a-b, 4d-e and 5a-b and Supplementary Figs 5d, 6b, 7a-b, 7d, 7f, 8a-e, 9a-e, 10a-b and 11d-i are provided as a Source Data file. The previously published protein structures were retrieved from Protein Data Bank (http://www.rcsb.org), URLs to these structures are provided in the related figure legends.

Field-specific reporting

Please select the one below that is the best fit for your research. If you are not sure, read the appropriate sections before making your selection.

☒ Life sciences ☐ Behavioural & social sciences ☐ Ecological, evolutionary & environmental sciences

For a reference copy of the document with all sections, see nature.com/documents/nr-reporting-summary-flat.pdf

Life sciences study design

All studies must disclose on these points even when the disclosure is negative.

Sample size	No sample-size calculations were performed. Three independent repeats for the ATPase, FP, ubiquitination, replication fork regression and HDX experiments were performed. These repeats produced similar results, indicating that the measurements were reliable and three repeats were sufficient.
Data exclusions	A small number of measurements (less than 5% of the total measurements) in the FP experiments that are clear outliers in the repeated experiments were excluded from the analysis. They are probably caused by instrument instability.
Replication	Three independent repeats were performed for the ATPase, FP, ubiquitination, replication fork regression and HDX experiments, they agree well.
Randomization	We did not use randomization in our study. The repeats of our experiments mentioned above produced consistent data and revealed significant differences between samples, supporting our conclusions.
Blinding	We did not use blinding in our study. The repeats of our experiments mentioned above produced consistent data and revealed significant differences between samples, supporting our conclusions.

Reporting for specific materials, systems and methods

We require information from authors about some types of materials, experimental systems and methods used in many studies. Here, indicate whether each material, system or method listed is relevant to your study. If you are not sure if a list item applies to your research, read the appropriate section before selecting a response.

Materials & experimental systems

n/a	Involved in the study	
☐	☒	Antibodies
☒	☐	Eukaryotic cell lines
☒	☐	Palaeontology and archaeology
☒	☐	Animals and other organisms
☒	☐	Human research participants
☒	☐	Clinical data
☒	☐	Dual use research of concern

Methods

n/a	Involved in the study	
☒	☐	ChIP-seq
☒	☐	Flow cytometry
☒	☐	MRI-based neuroimaging

Antibodies

Antibodies used	Anti-flag (a8592, Sigma-Aldrich) , anti-HA (ab18181, Abcam), anti-ubiquitin (PD41, Abcam), anti-PCNA (gift from Dr. Bruce Stillman at Cold Spring Harbor Laboratory), anti-Pgk1 (22C5D8, Invitrogen), anti-histone H3 (ab46765, Abcam), anti-TAP (P1291, Sigma-Aldrich), anti-Histag (ab1269, abcam), anti-Rad5 (yS-15, Santa Cruz Biotechnology).
Validation	The anti-PCNA anti-body has been validated by Ulrich and Davies (In vivo detection and characterization of sumoylation targets in Saccharomyces cerevisiae. Methods Mol Biol 497, 81-103, doi:10.1007/978-1-59745-566-4_6 (2009)). The anti-TAP (P1291, Sigma-Aldrich) anti-body is produced in rabbit, it has been used in a number of previous studies to detect TAP-tagged proteins by western blotting, for instance in the study by Mayer et al (The spt5 C-terminal region recruits yeast 3' RNA cleavage factor I. Mol Cell Biol. 32 (7): 1321-31, doi: 10.1128/MCB.06310-11). The rest of anti-bodies used in this study have been validated by the commercial providers. The following information is taken from the providers' websites. The biological source of the Anti-flag (a8592, Sigma-Aldrich), anti-HA (ab18181, Abcam), anti-ubiquitin (PD41, Abcam) and anti-Pgk1 (22C5D8, Invitrogen) anti-bodies is mouse; of the anti-histone H3 (ab46765, Abcam) anti-body is rabbit; of the anti-Histag (ab1269, abcam) and anti-Rad5 (yS-15, Santa Cruz Biotechnology) anti-bodies is goat. All these antibodies are suitable for western blotting.
