# Supplementary figures and images for: Structural basis for the multi-activity factor Rad5 in replication stress tolerance
Source: Nat Commun. 2021 Jan 12;12:321. doi: 10.1038/s41467-020-20538-w (PMC7804152; doi:10.1038/s41467-020-20538-w)

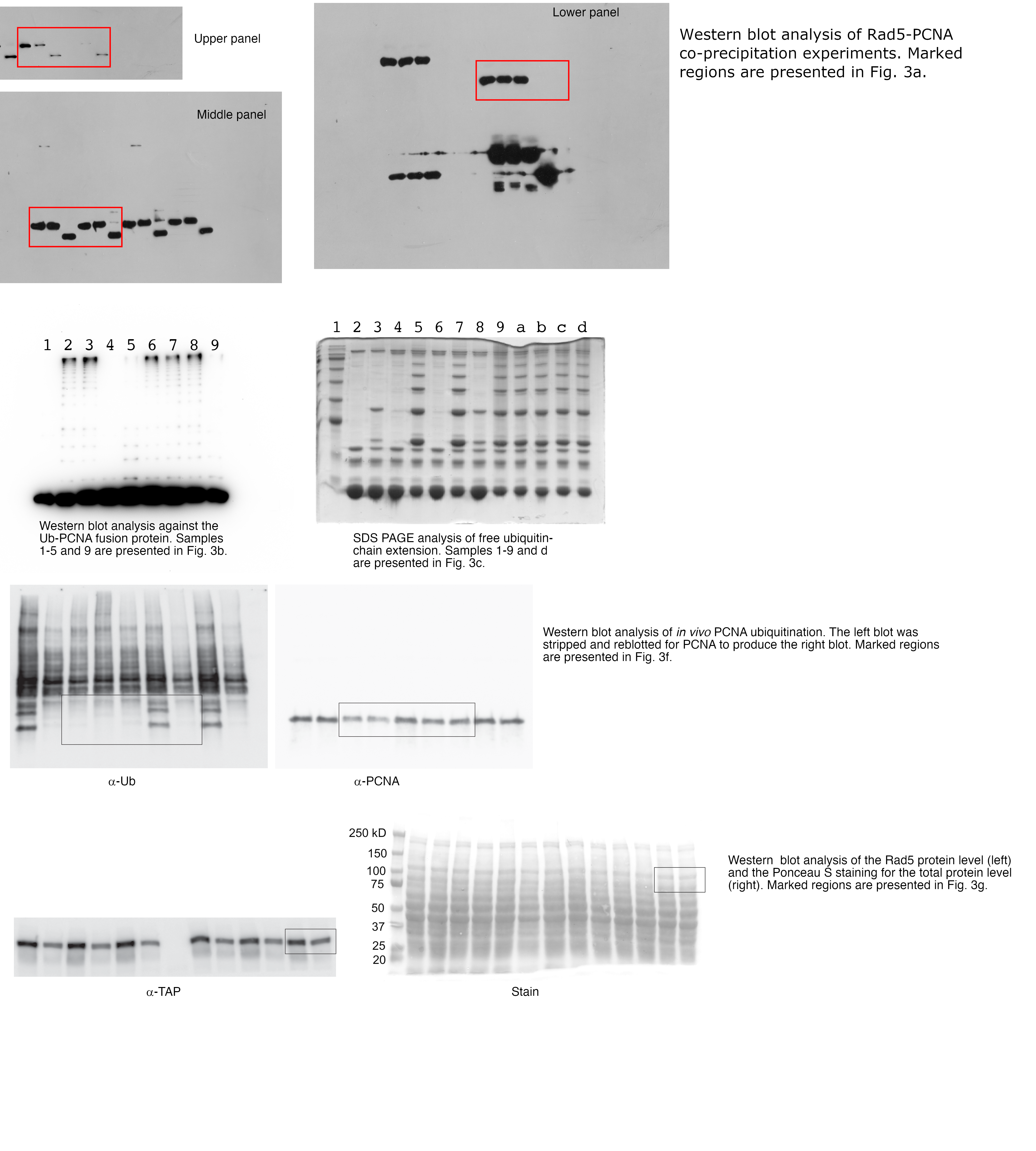

Supplement: Supplementary file 4 — Source Data [file 41467_2020_20538_MOESM4_ESM.zip › Source_data/Fig3_source_data.tiff]

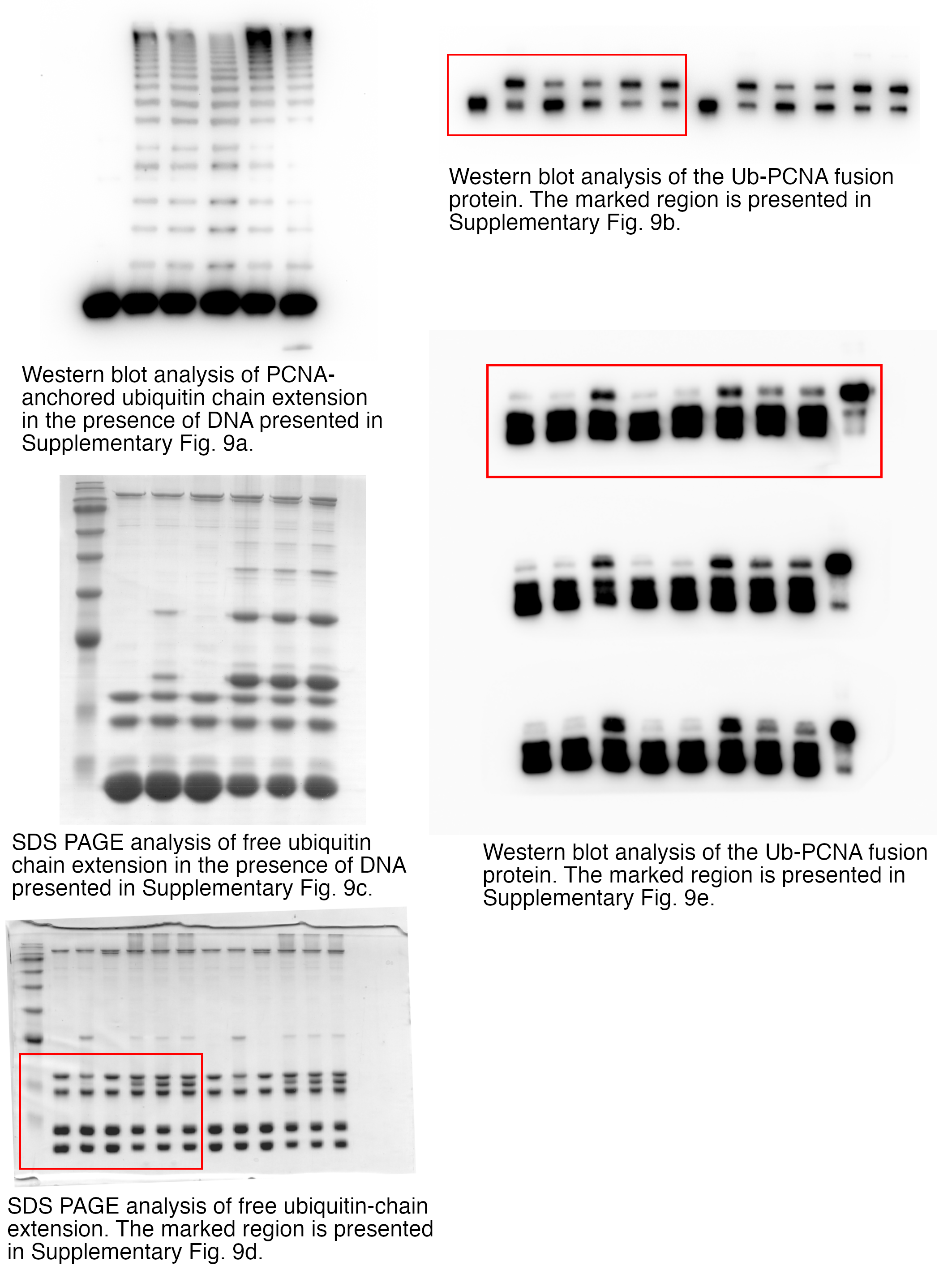

Supplement: Supplementary file 4 — Source Data [file 41467_2020_20538_MOESM4_ESM.zip › Source_data/FigS9_source_data.tiff]

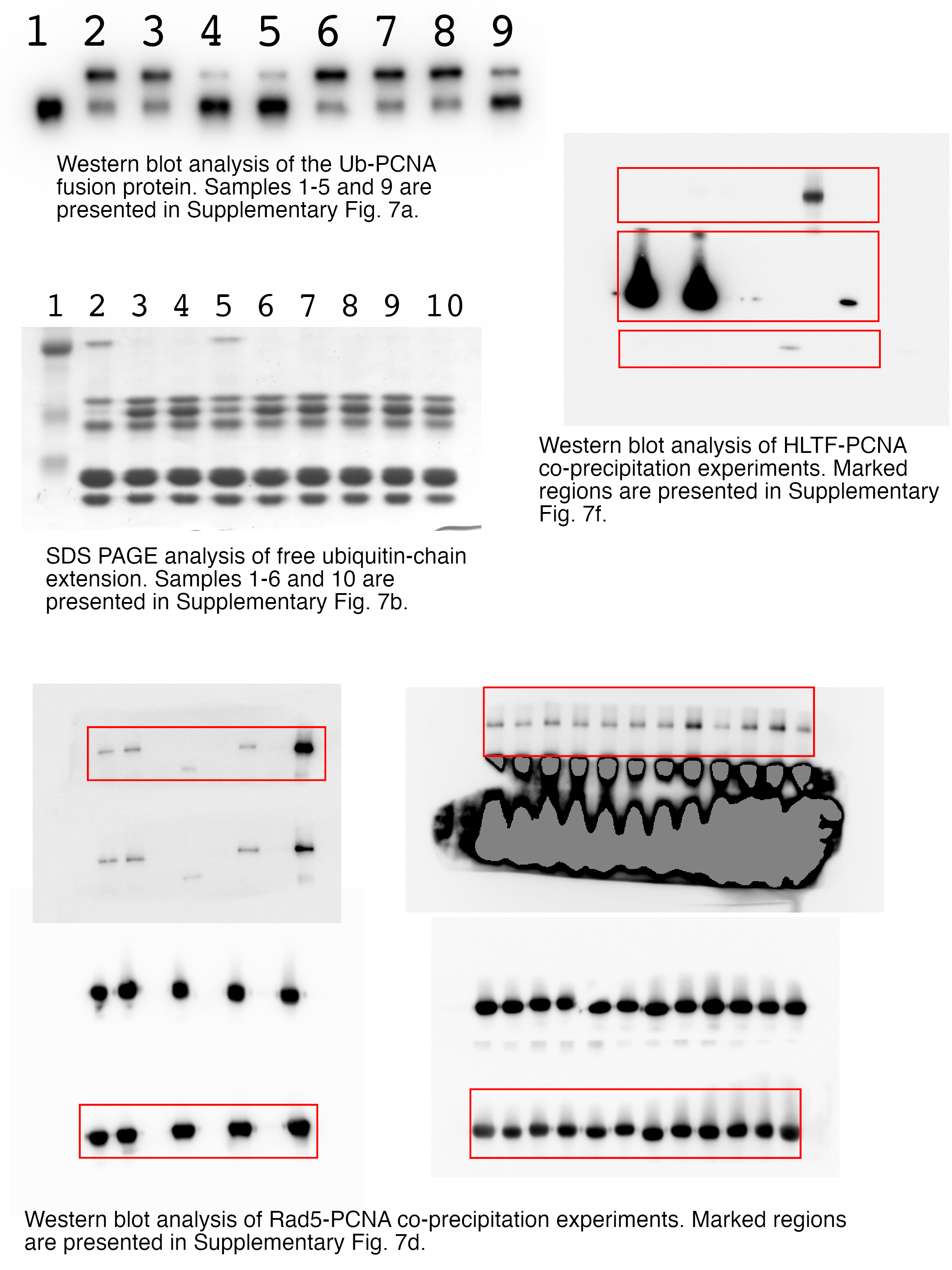

Supplement: Supplementary file 4 — Source Data [file 41467_2020_20538_MOESM4_ESM.zip › Source_data/FigS7_source_data.tiff]

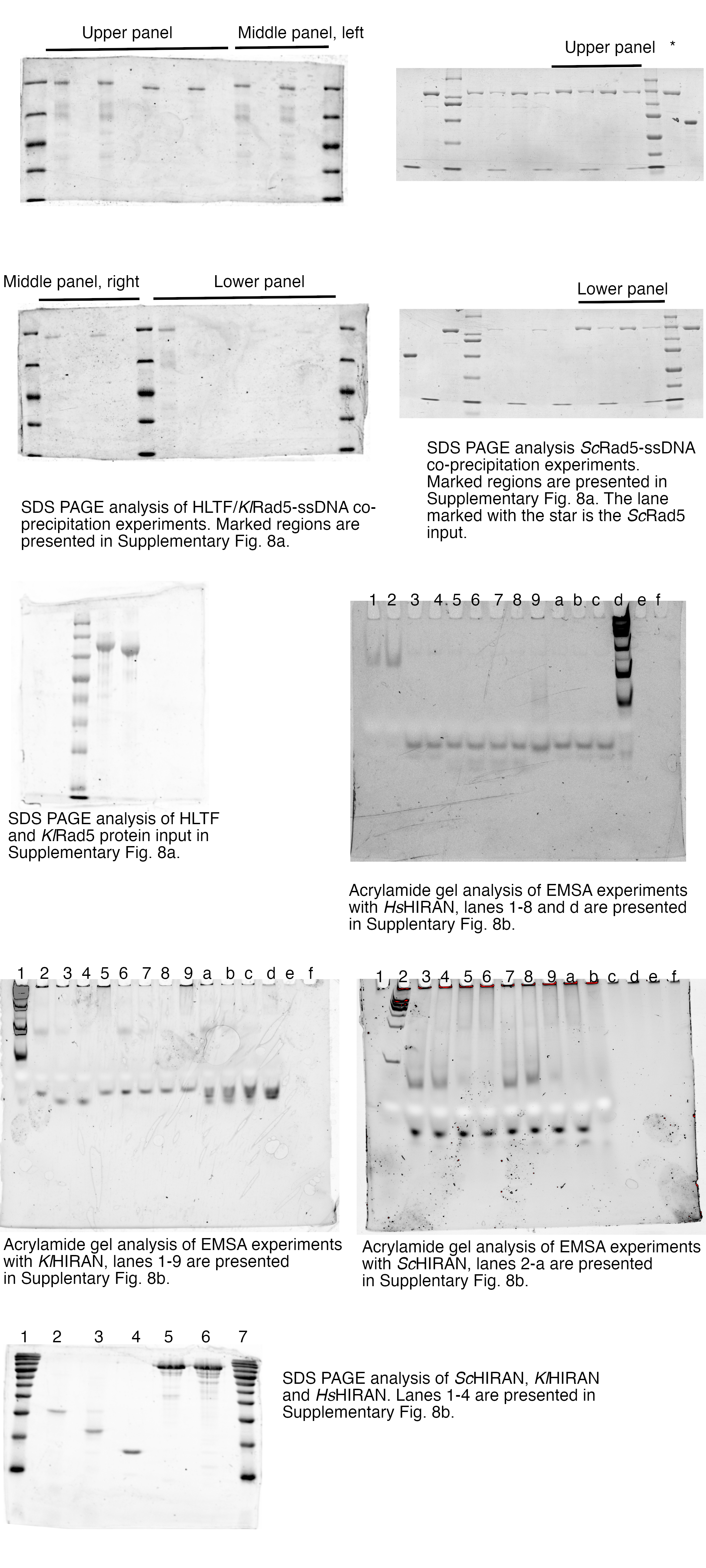

Supplement: Supplementary file 4 — Source Data [file 41467_2020_20538_MOESM4_ESM.zip › Source_data/FigS8_source_data.tiff]

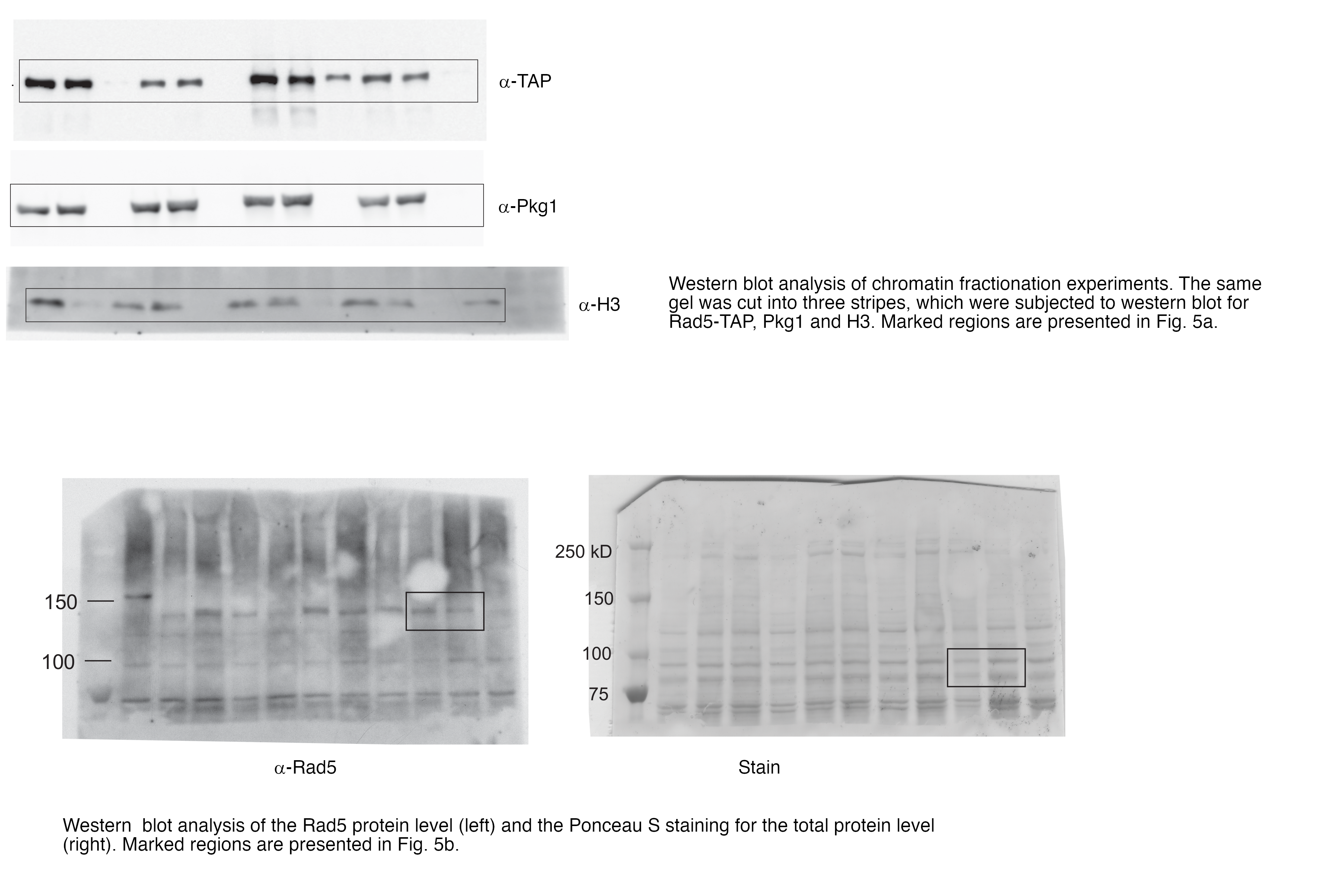

Supplement: Supplementary file 4 — Source Data [file 41467_2020_20538_MOESM4_ESM.zip › Source_data/Fig5_source_data.tiff]

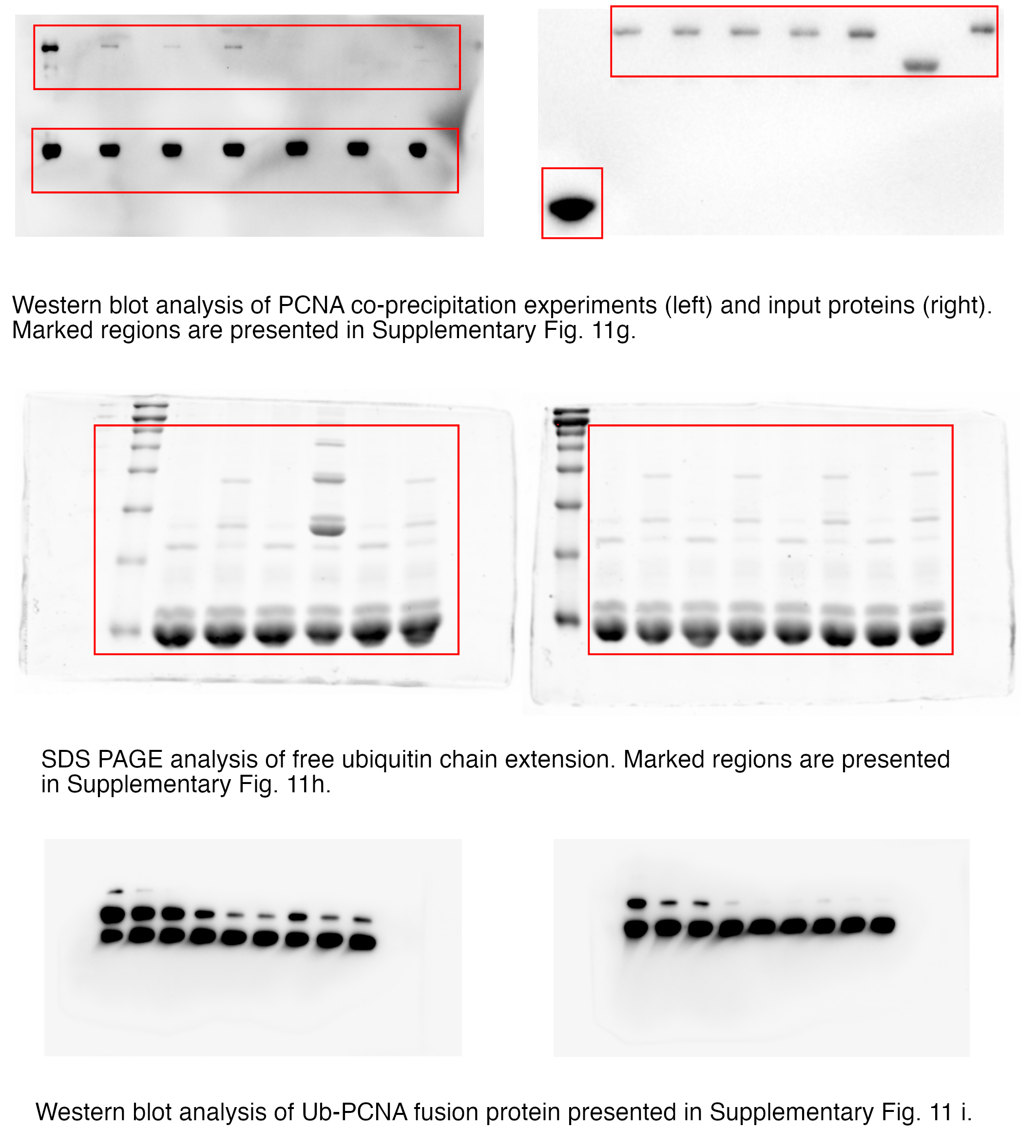

Supplement: Supplementary file 4 — Source Data [file 41467_2020_20538_MOESM4_ESM.zip › Source_data/FigS11_source_data.tiff]

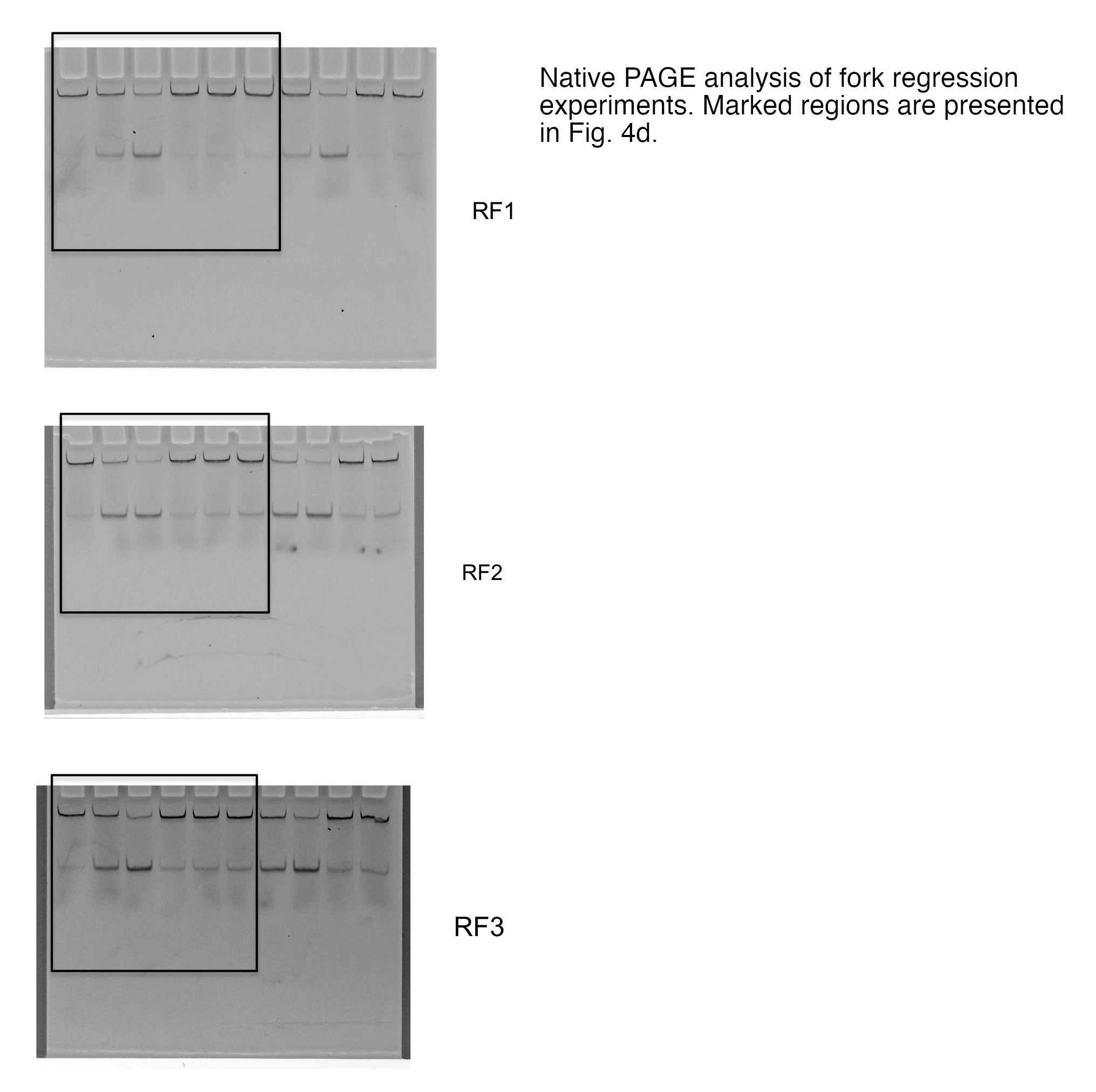

Supplement: Supplementary file 4 — Source Data [file 41467_2020_20538_MOESM4_ESM.zip › Source_data/Fig4_source_data.tiff]

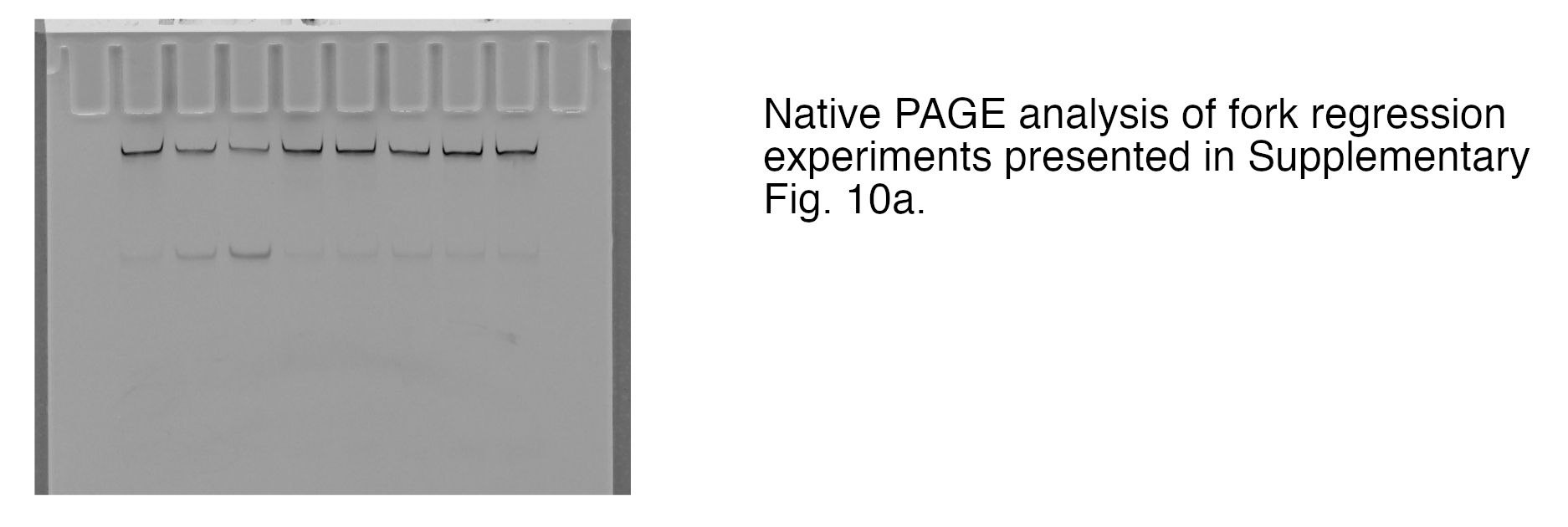

Supplement: Supplementary file 4 — Source Data [file 41467_2020_20538_MOESM4_ESM.zip › Source_data/FigS10_source_data.tiff]
